# Quantitative evaluation of Scout Accelerated Motion Estimation and Reduction (SAMER) MPRAGE for morphometric analysis of brain tissue in patients undergoing evaluation for memory loss

**DOI:** 10.1016/j.neuroimage.2024.120865

**Published:** 2024-09-28

**Authors:** Nelson Gil, Azadeh Tabari, Wei-Ching Lo, Bryan Clifford, Min Lang, Komal Awan, Kyla Gaudet, Daniel Nicolas Splitthoff, Daniel Polak, Stephen Cauley, Susie Y. Huang

**Affiliations:** a Department of Radiology, Massachusetts General Hospital, Boston, MA, USA; b Harvard Medical School, Boston, MA, USA; c Siemens Medical Solutions USA, Boston, MA, USA; d Siemens Healthineers, Erlangen, Germany; e Department of Radiology, A. A. Martinos Center for Biomedical Imaging, Massachusetts General Hospital, Charlestown, MA, USA

**Keywords:** Volumetric brain MRI, Motion correction, Morphometry, Memory loss

## Abstract

**Background:**

Three-dimensional (3D) T1-weighted MRI sequences such as the magnetization prepared rapid gradient echo (MPRAGE) sequence are important for assessing regional cortical atrophy in the clinical evaluation of dementia but have long acquisition times and are prone to motion artifact. The recently developed Scout Accelerated Motion Estimation and Reduction (SAMER) retrospective motion correction method addresses motion artifact within clinically-acceptable computation times and has been validated through qualitative evaluation in inpatient and emergency settings.

**Methods:**

We evaluated the quantitative accuracy of morphometric analysis of SAMER motion-corrected compared to non-motion-corrected MPRAGE images by estimating cortical volume and thickness across neuroanatomical regions in two subject groups: (1) healthy volunteers and (2) patients undergoing evaluation for dementia. In part (1), we used a set of 108 MPRAGE reconstructed images derived from 12 healthy volunteers to systematically assess the effectiveness of SAMER in correcting varying degrees of motion corruption, ranging from mild to severe. In part (2), 29 patients who were scheduled for brain MRI with memory loss protocol and had motion corruption on their clinical MPRAGE scans were prospectively enrolled.

**Results:**

In part (1), SAMER resulted in effective correction of motion-induced cortical volume and thickness reductions. We observed systematic increases in the estimated cortical volume and thickness across all neuroanatomical regions and a relative reduction in percent error values compared to reference standard scans of up to 66 % for the cerebral white matter volume. In part (2), SAMER resulted in statistically significant volume increases across anatomical regions, with the most pronounced increases seen in the parietal and temporal lobes, and general reductions in percent error relative to reference standard clinical scans.

**Conclusion:**

SAMER improves the accuracy of morphometry through systematic increases and recovery of the estimated cortical volume and cortical thickness following motion correction, which may affect the evaluation of regional cortical atrophy in patients undergoing evaluation for dementia.

## 1. Introduction

MRI is one of the most powerful neuroimaging modalities in clinical practice due to its ability to precisely characterize gray and white matter anatomy and pathology. However, MRI is limited in practice by artifacts introduced due to patient motion, which can obscure important structural details and result in costly non-diagnostic scans (Andre et al., 2015; Munn et al., 2015; Havsteen et al., 2017; Slipsager et al., 2020). One of the most motion-prone exam types is volumetric brain MRI, which quantifies characteristics such as cortical volume and thickness and represents an important aid in the diagnosis of neurodegenerative diseases or dementia (Lehmann et al., 2010; Ries et al., 2008; Raji et al., 2019). Specifically, high-resolution T1-weighted sequences such as the T1-weighted magnetization prepared rapid gradient echo (MPRAGE) sequence (Mugler and Brookeman, 1990) are a standard part of MRI protocols to evaluate memory loss and serve as the basis for gray and white matter volumetric measurements; nevertheless, it is especially prone to long acquisition times and motion artifacts (Mugler and Brookeman, 1991). Furthermore, scans from patients with mild cognitive impairment or Alzheimer’s dementia, common in the elderly population frequently evaluated by memory loss protocols, are especially likely to display motion artifacts (Haller et al., 2014; Savalia et al., 2017). Accurate correction of motion artifacts would therefore lead to improved and more cost-effective clinical care.

Efforts to mitigate motion artifacts generally center on applying motion correction algorithms. These approaches involve specialized image acquisition and reconstruction techniques that can measure and correct for patient motion prospectively (during acquisition) or retrospectively (after acquisition) in the image domain or the corresponding k-space data (Godenschweger et al., 2016). For example, some prospective motion-correction methods rely on real-time tracking of head motion (White et al., 2010) to adjust MRI pulse sequences during image acquisition. Other approaches involve specific ways of traversing k-space during image reconstruction that oversample portions of the data to reduce motion artifact (e.g. PROPELLER) (Pipe et al., 2014; Pipe, 1999). Navigator-based approaches (Sarlls et al., 2018) apply additional radiofrequency excitations to track anatomic motion, yielding information that can be used to correct for motion during the acquisition. In addition, navigator-based approaches may be combined with other techniques, such as those that utilize optical systems to measure motion (Norbeck et al., 2021).

Retrospective motion correction approaches may be applied to the acquired data that can correct complex deformations (Cheng et al., 2012), but these may be computationally intensive, making them less practical in clinical settings unless suitably optimized. Long image reconstruction times may be potentially mitigated using pre-trained machine learning or deep learning models (Pawar et al., 2020; Al-Masni et al., 2022; Nalini et al., 2023) that are currently under investigation.

Practical approaches to mitigating motion within scans include shortening MRI acquisition times, which will in many cases intrinsically reduce motion artifact. Accelerated image acquisition strategies that have been applied specifically to the MPRAGE sequence include Wave Controlled Aliasing in Parallel Imaging (Wave-CAIPI) (Polak et al., 2018) and compressed sensing (CS) (Mussard et al., 2020; Ferraro et al., 2022). While shortening acquisition time is limited by the need for clinically acceptable signal-to-noise ratio in the images (Davis et al., 1996), strategies such as Wave-CAIPI-accelerated MPRAGE have been shown to achieve nearly an order of magnitude acceleration. Wave-CAIPI MPRAGE has been validated clinically against standard MPRAGE and shown to provide equivalent assessments of cortical volume and thickness, offering a promising approach for mitigating motion in patients undergoing dementia evaluation (Longo et al., 2020; Sakurama et al., 2021).

Ultimately, accelerated MRI acquisitions may work synergistically with motion correction algorithms. One such promising motion correction approach is the recently developed Scout-Accelerated Motion Estimation and Correction (SAMER) method (Polak et al., 2022; Polak et al., 2023). SAMER uses an initial 3–5 second scout image as a guide to adjust for motion-corrupted k-space trajectories during the acquisition. Initial clinical validation of the SAMER approach has shown that SAMER improves the diagnostic image quality of clinical brain MRI examinations performed in the inpatient and emergency settings based on qualitative evaluation by expert neuroradiologists (Lang et al., 2023). Preliminary evidence suggests that applying SAMER to standard MPRAGE (a “SAMER-MPRAGE” sequence) can successfully correct for motion artifact and provide quantitative cortical volume and thickness estimates comparable to those obtained with standard MPRAGE (Lo et al., 2022; Gil et al., 2023).

**Fig. 1.**
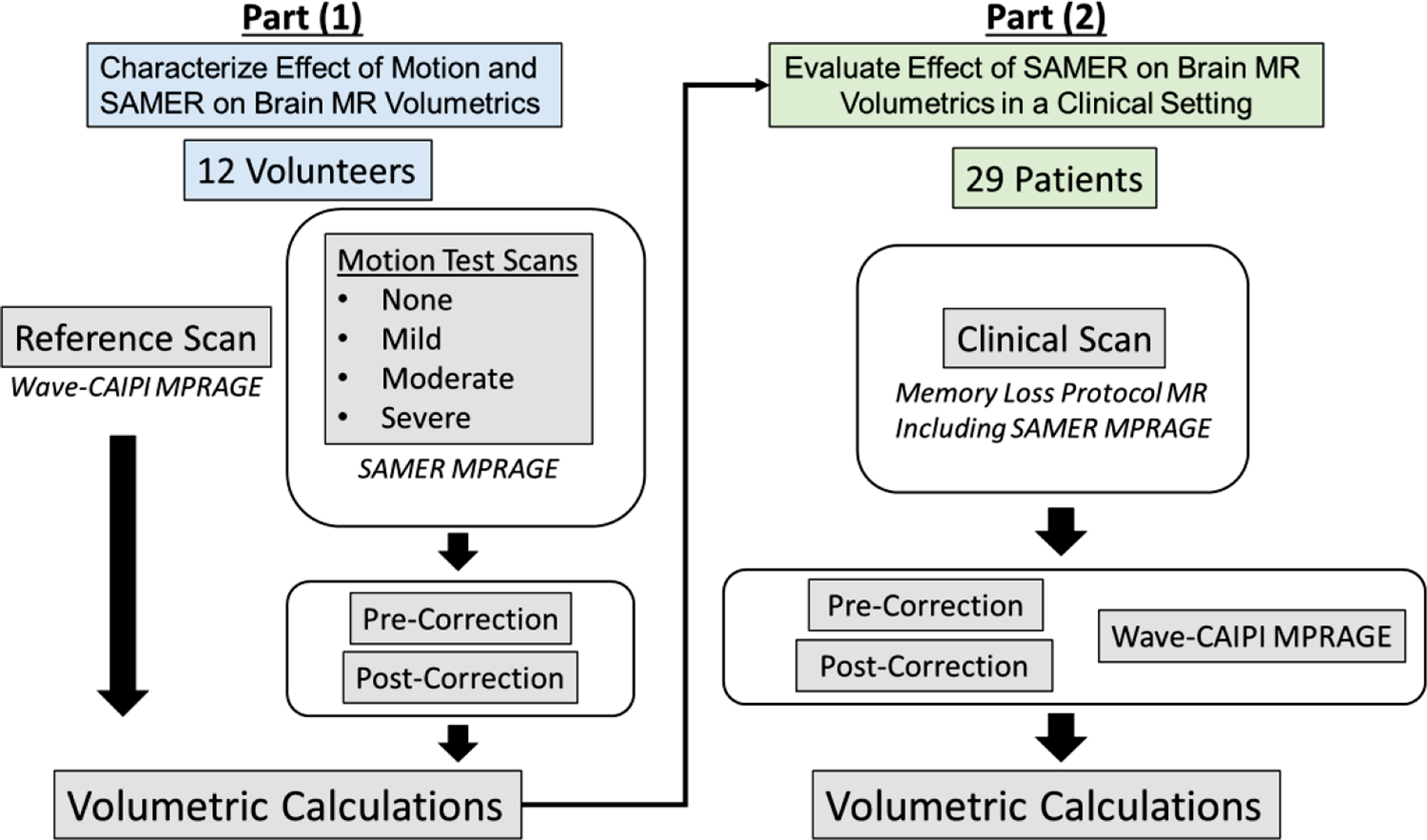
Schematic diagram showing the design of this two-part study involving both healthy volunteers and patients. Volumetrics obtained from the healthy volunteers provided guidance on how to interpret the results from SAMER applied in actual patients.

In this work, we conducted a systematic two-part study to characterize the effectiveness of SAMER in improving the accuracy of MPRAGE-derived volumetric measurements in both a controlled experimental setting on volunteers and a real-life outpatient clinical setting in patients undergoing evaluation for memory loss (Fig. 1). In part (1), we performed SAMER reconstruction of MPRAGE images derived from healthy adult volunteers who were instructed to move with varying degrees of head motion to characterize the degree to which SAMER can correct motion-induced errors in different anatomical regions of the brain as assessed by quantitative volumetric analyses. In part (2), we deployed SAMER in a real-world outpatient clinical setting in patients undergoing evaluation for memory loss. The overall rationale for our study design was to use the observed differences in volumetric measurements of the volunteers from part (1) to guide the interpretation of results observed in patients in part (2). Our work provides key insights into the performance of quantitative volumetric analyses following motion correction by SAMER, as applied to scenarios with well-characterized degrees of motion severity, and demonstrates the value of SAMER motion correction in improving clinically-relevant quantitative measurements of cortical volume and thickness in patients undergoing evaluation for memory loss.

## 2. Methods

### 2.1. Design and subjects

All components of this study were compliant with the Health Insurance Portability and Accountability Act and were approved by the Mass General Brigham institutional review board. Fig. 1 presents a schematic diagram outlining the overall study design. Part (1) involved a controlled experiment using healthy volunteers to characterize the effect of varying degrees of motion artifact (none, mild, moderate, and severe) on volumetric measurements obtained from SAMER-MPRAGE scans compared to a Wave-CAIPI MPRAGE reference scan. The Wave-CAIPI MPRAGE scan was chosen as the reference due to its short acquisition time (see details under **Scan acquisition**) and robustness to motion. Wave-CAIPI MPRAGE has been validated for accurate quantitative brain volumetry in both volunteers (Longo et al., 2020) and dementia patients (Elliott et al., 2023). Part (2) involved a prospective clinical validation study in patients undergoing evaluation for dementia with memory loss protocol brain MRIs that included the Wave-CAIPI MPRAGE research sequence as the reference and the SAMER-MPRAGE research sequence added to the end of the protocol. The differences in volumetric measurements obtained before and after SAMER motion correction were compared to those obtained from the Wave-CAIPI MPRAGE scan. The results of part (1) helped to guide the interpretation of the results in the clinical study of part (2), in that we were able to treat the Wave-CAIPI MPRAGE scans in both cases as reference standards against which to measure the effect of motion correction.

### 2.2. Part (1): Healthy volunteer study

12 healthy adult volunteers were recruited between October and November 2022 to participate in part (1) of this study (Table 1). Written informed consent was obtained prior to MRI as per standard protocols in accordance with the Declaration of Helsinki. All scans were acquired on a 3T system (MAGNETOM Skyra, Siemens Healthcare, Erlangen, Germany) using a 20-channel head coil.

Each volunteer underwent five different in vivo MPRAGE scans at *R* = 4-fold acceleration. A Wave-CAIPI-MPRAGE sequence was performed first following instructions to the subject to remain as still as possible and served as the reference “motion-free” standard. The other four MPRAGE scans corresponded to acquisitions performed with a SAMER-MPRAGE research sequence corresponding to four different motion states: “no”, “mild”, “moderate”, and “severe”, as defined in a five-point scale for motion corruption introduced in prior work (Lang et al., 2023) (Supplemental Table 1). Before the scan began, subjects were shown examples of head movements that corresponded to “mild”, “moderate”, and “severe” motion. Once the scan began, subjects were given real-time feedback until a desired level of motion corruption in their image was achieved. Subjects were purposely not instructed to move in any specific way (e.g. head rotation, repeated motions) to sample a broad set of motion trajectories. Studies corresponding to “minimal” motion on the scale were not performed. All images were reviewed and graded for motion by a board-certified neuroradiologist during the scan session.

Motion-corrupted scans underwent motion correction with SAMER, producing four additional reconstructions corresponding to each motion state: “no”, “mild”, “moderate”, and “severe” motion. In total, each volunteer generated nine reconstructed image series: Wave-CAIPI MPRAGE and SAMER-MPRAGE pre- and post-motion correction for each of the four motion states. This process resulted in 108 effective total number of MRI reconstructions analyzed across the 12 volunteers. The sequence parameters used for Wave-CAIPI MPRAGE and SAMER-MPRAGE are listed in Table 2.

### 2.3. Quantitative motion metric and regression analysis

For the volunteer study, we utilized a quantitative motion metric adapted from prior work characterizing volumetric changes due to head motion, treating the brain as a sphere centered at the isocenter with a radius *r* of 64 mm and modeling motion as a combination of translations and rotations (affine transformations) (Reuter et al., 2015). For a motion tracking experiment with *n* timepoints, the quantitative motion metric (in mm/s) was a root mean square (RMS) deviation defined for sequential rigid affine transformations *T*_*i,i* +__*1*_ as:

$$\text{RMS}=\frac{1}{n•\tau_{R}}\overset{n-1}{\underset{i=0}{\sum }}{\text{RMS}}_{r}\left(T_{i,i+1}\right)$$

Here, *τ*_*R*_ = 2.3 s, which is the repetition time (TR) value of the MPRAGE sequences we utilized (Table 2), and *RMS*_*r*_ is the RMS deviation for a spherical volume with radius *r*, which for a given translation vector *t* and rotation matrix *M* is described by:

$${\text{RMS}}_{r}=\sqrt{\frac{1}{5}r^{2}\text{tr}\left[{\left(M-I\right)}^{T}(M-I)\right]+t^{T}t}$$

Where *I* is the identity matrix.

We correlated the quantitative motion metrics for the total brain for all volunteers for the scans of each motion state with their corresponding percent error values relative to Wave-CAIPI MPRAGE reference scans and performed linear regression analysis, which we additionally stratified by pre- and post-SAMER-correction status for each of the four motion states (no, mild, moderate, and severe motion). In addition, we performed a regression analysis of the percent change in volume and thickness of the total brain versus the quantitative motion metric for the “no motion” state.

### 2.4. Part (2): Patient study

We prospectively enrolled 29 adult patients undergoing clinical evaluation for dementia who were scheduled for memory loss protocol brain MRI between January and August 2023 (Table 1). Verbal consent was obtained at the time of MR imaging, as written consent was waived by the IRB for this minimal risk study. Patients obtained Wave-CAIPI MPRAGE and SAMER-MPRAGE scans, all acquired on a 3T system (MAGNETOM Prisma, Siemens Healthcare, Erlangen, Germany) using a 20-channel head coil. Patients undergoing brain MR imaging as part of the clinical workup for memory loss or suspected neurodegenerative disease were included. The scans included patients being imaged for objective or subjective complaints of memory loss, including patients in whom there was concern for dementia (15 of 29) or underlying neurological disorders, such as traumatic brain injury (Table 1). We retrospectively evaluated patients whose SAMER pre-correction scans visually appeared motion-degraded to at least a “minimal” degree on initial inspection, based on a previously validated motion-corruption scale (Lang et al., 2023). Exclusion criteria were similar to those for routine clinical MR imaging. One patient did not have a reference Wave-CAIPI MPRAGE scan available, but still had SAMER pre- and post-motion-correction sequences and was still included in comparisons that only involved pre- and post-motion-correction sequences.

Each patient underwent a clinical memory loss protocol MRI that contained a Wave-CAIPI MPRAGE research sequence as the first scan of the examination and a SAMER-MPRAGE research sequence added to the end of the examination, using the same sequence parameters as in part (1) (Table 2). No additional instructions were given to patients. Motion estimation and image reconstruction were performed with an online reconstruction directly on the scanner. The raw k-space data from the SAMER-MPRAGE images was saved and extracted from the scanner within 48 h of acquisition. The SAMER framework (Polak et al., 2022) was then retrospectively applied to the extracted SAMER-MPRAGE raw data for motion correction.

### 2.5. Volumetric calculations and statistical analysis

#### Part (1):

For the nine sets of reconstructed images from each of the 12 volunteers, the longitudinal FreeSurfer pipeline (Fischl, 2012; Reuter et al., 2012) without any software dependencies was used to calculate cortical volumes for 11 brain regions (frontal, parietal, temporal, occipital lobes, cingulate gyrus, insula, hippocampus, basal ganglia, brain stem, cerebellum, cerebral white matter (WM)) and thickness for 6 brain regions (frontal, parietal, temporal, occipital lobes, cingulate gyrus, insula). All brain regions were automatically segmented by FreeSurfer before and after motion correction.

The statistical significance of SAMER motion correction was assessed using two-tailed paired Wilcoxon signed rank tests comparing the cortical thickness and volume across each anatomical region for uncorrected versus corrected scans corresponding to each of the four motion states, as enumerated in Table 3. The p-value threshold for statistical significance was set at <0.0011 for cortical volume and <0.0021 for cortical thickness by applying Bonferroni correction based on the number of anatomical regions being compared, multiplied by the number of motion states being compared.

In addition, cortical volumes and thicknesses for each region were averaged across all volunteers. Percent-error for the averaged cortical volume and thickness was calculated, treating Wave-CAIPI-MPRAGE results as the reference standard:

$$\text{PercentError}=100*\frac{|\text{AveragedVolumeOrThickness}-\text{Reference}|}{\text{Reference}}$$

#### Part (2):

FreeSurfer was used to calculate the cortical volume and thickness for the same 11 and 6 anatomical regions as in part (1), respectively. These volumetric calculations were carried out for SAMER-MPRAGE sequences, both before and after motion correction. In addition, as a “reference standard”, we performed volumetric calculations on the Wave-CAIPI MPRAGE images for each patient. The percent difference in cortical volume and thickness after motion correction was computed and averaged over all 29 patients for post-correction SAMER-MPRAGE scans relative to their pre-correction counterparts. In addition, the percent difference was calculated for both pre- and post-correction SAMER-MPRAGE scans relative to the Wave-CAIPI MPRAGE reference scan.

Like in part (1), the statistical significance of SAMER motion correction was assessed using two-tailed paired Wilcoxon signed rank tests comparing the cortical thickness and volume across each anatomical region for uncorrected versus corrected scans as well as Wave-CAIPI MPRAGE scans, as enumerated in Table 4. The p-value threshold was set at <0.0014 for cortical volume and <0.0024 for cortical thickness, corresponding to the number of anatomical regions and number of scan types being compared.

## 3. Results

### 3.1. Part (1): Effect of SAMER motion correction on estimated cortical volume and cortical thickness in healthy volunteers

Twelve healthy volunteers (mean age 48.3 ± 13 years, 4 women; Table 1) participated in the systematic evaluation of motion on quantitative volumetric analyses. Using segmentations automatically derived from FreeSurfer (Fischl, 2012), we systematically calculated the cortical volumes and thicknesses for 12 volunteers (Table 3) across 11 (for volume) or 6 (for thickness) neuroanatomical areas. The calculations were performed on SAMER-MPRAGE scans undergoing four levels of deep-breathing-induced motion corruption: “no”, “mild”, “moderate”, and “severe”, both before and after applying motion correction with SAMER. In addition, we also performed calculations on a “reference standard” Wave-CAIPI MPRAGE scan. Calculations were successfully completed on 101/108 (~94 %) reconstructed images; the 7 calculations that did not complete were due to either severe (6 cases) or moderate (1 case) motion artifact before motion correction that prevented adequate segmentation of anatomical regions. Applying SAMER motion correction resulted in successful computation of volumes and thicknesses for 6 of the 7 cases where FreeSurfer initially failed to provide morphometric results.

**Fig. 2.**
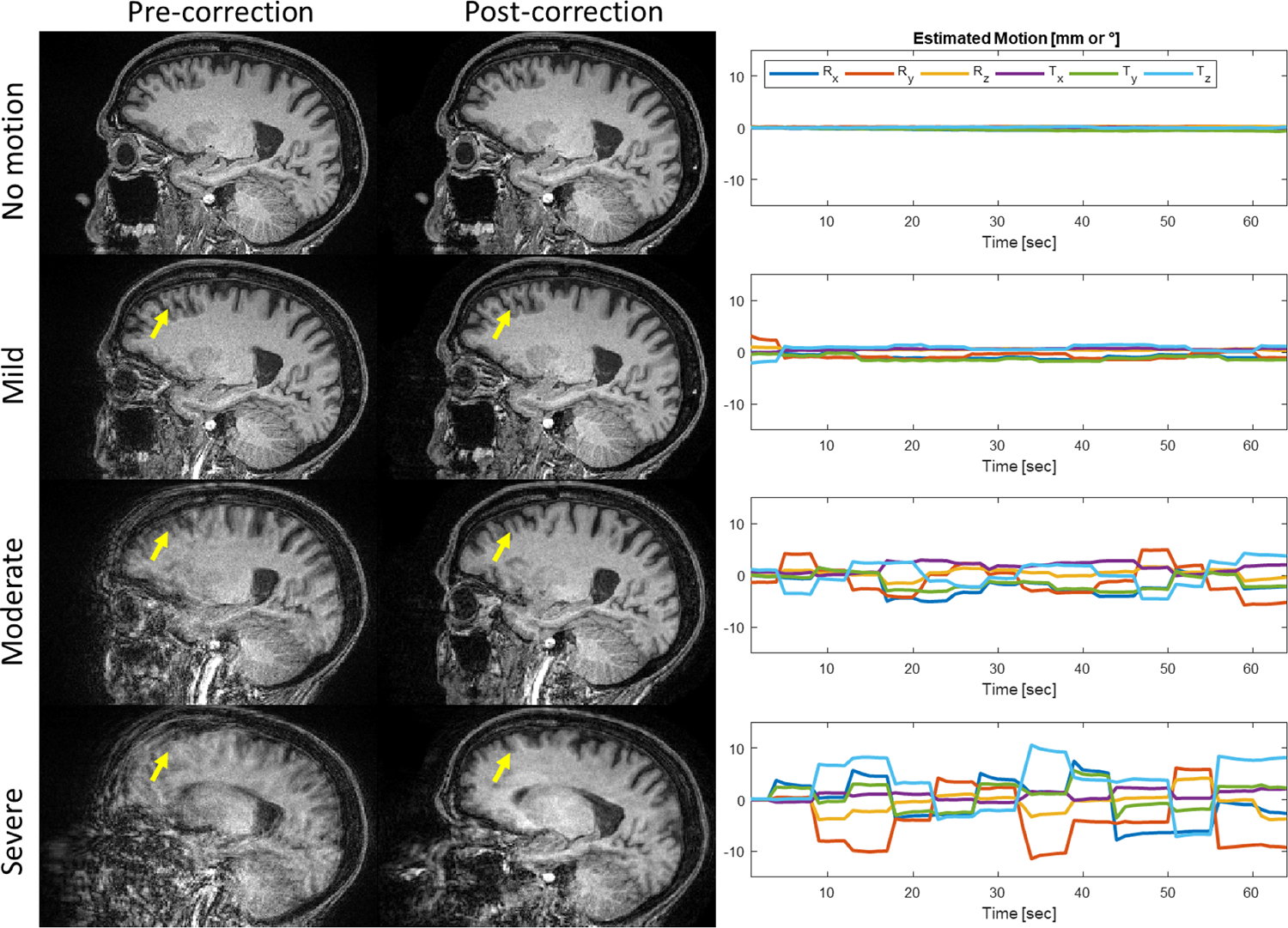
Example scans of a single subject representing four motion states: no motion, mild motion, moderate motion, and severe motion, along with SAMER motion correction and depiction of the estimated motion trajectory along translational (T_x_, T_y_, T_z_) and rotational (R_x_, R_y_, R_z_) degrees of freedom.

**Fig. 3.**
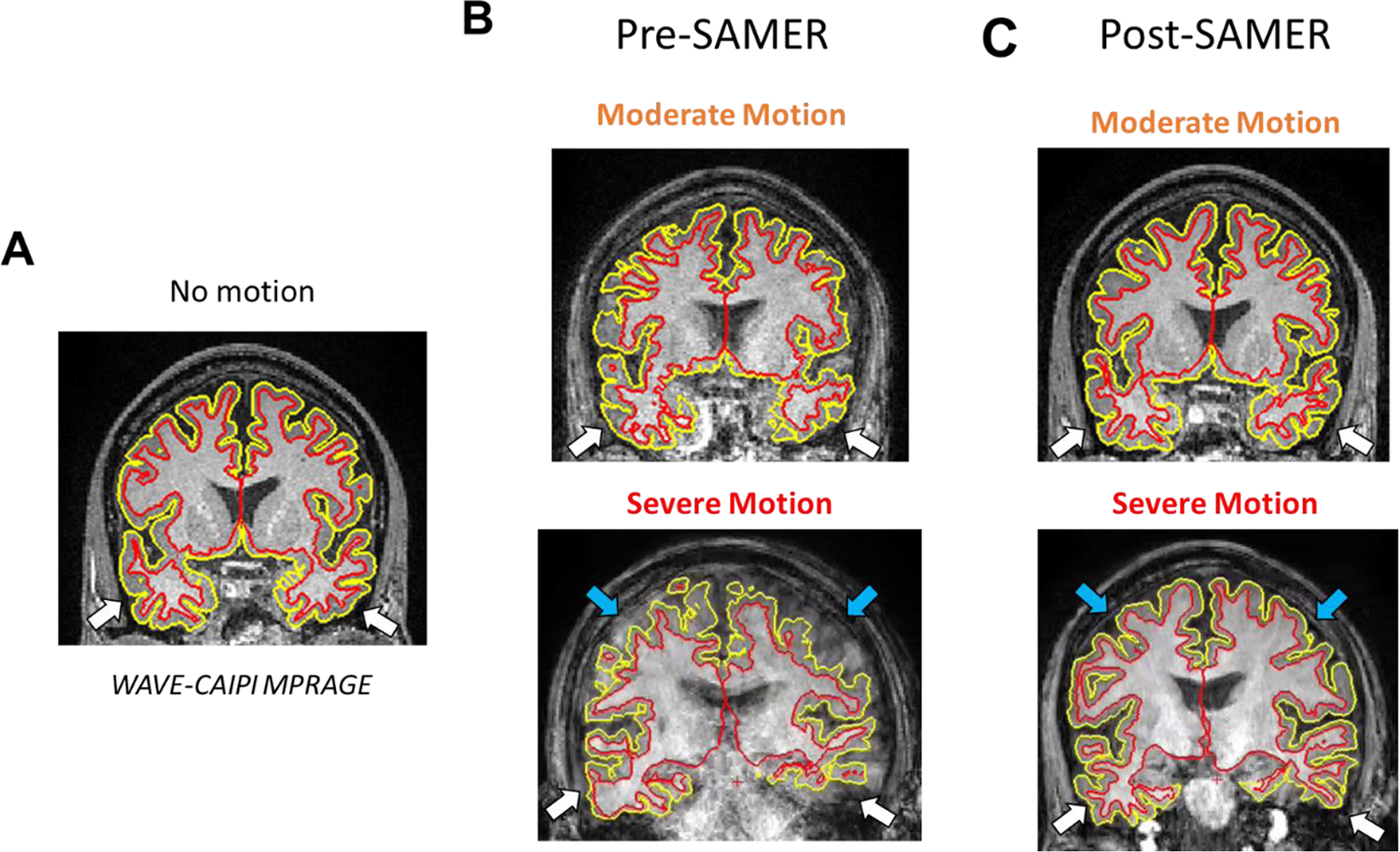
Representative Freesurfer segmentation on a coronal slice demonstrating the inner and outer cortical surface on a representative subject’s (A) no motion, (B) moderate motion before and after SAMER correction, and severe motion before and after SAMER correction scans. For example, for moderate and severe motion, the segmentation of the temporal lobes (white arrows) noticeably improves after motion correction and more closely resembles that of a scan without motion. In addition, for the severe motion case, the segmentation of the parietal lobes (blue arrows) appears greatly improved.

Representative cases illustrate the effect of mild, moderate, and severe degrees of motion on the appearance of brain tissue and subsequent correction with SAMER (Fig. 2): greater degrees of motion increasingly blur anatomical details, while SAMER can restore the ability to appreciate these details. In addition, examples of corresponding FreeSurfer segmentations for motion-corrupted images qualitatively demonstrate that, relative to a reference standard Wave-CAIPI MPRAGE sequence, FreeSurfer segmentations in motion-corrupted images are more irregular (Fig. 3), which would lead to erroneous estimations of cortical volume or thickness. Applying SAMER qualitatively resulted in the smoothness of the segmentations being restored.

**Fig. 4.**
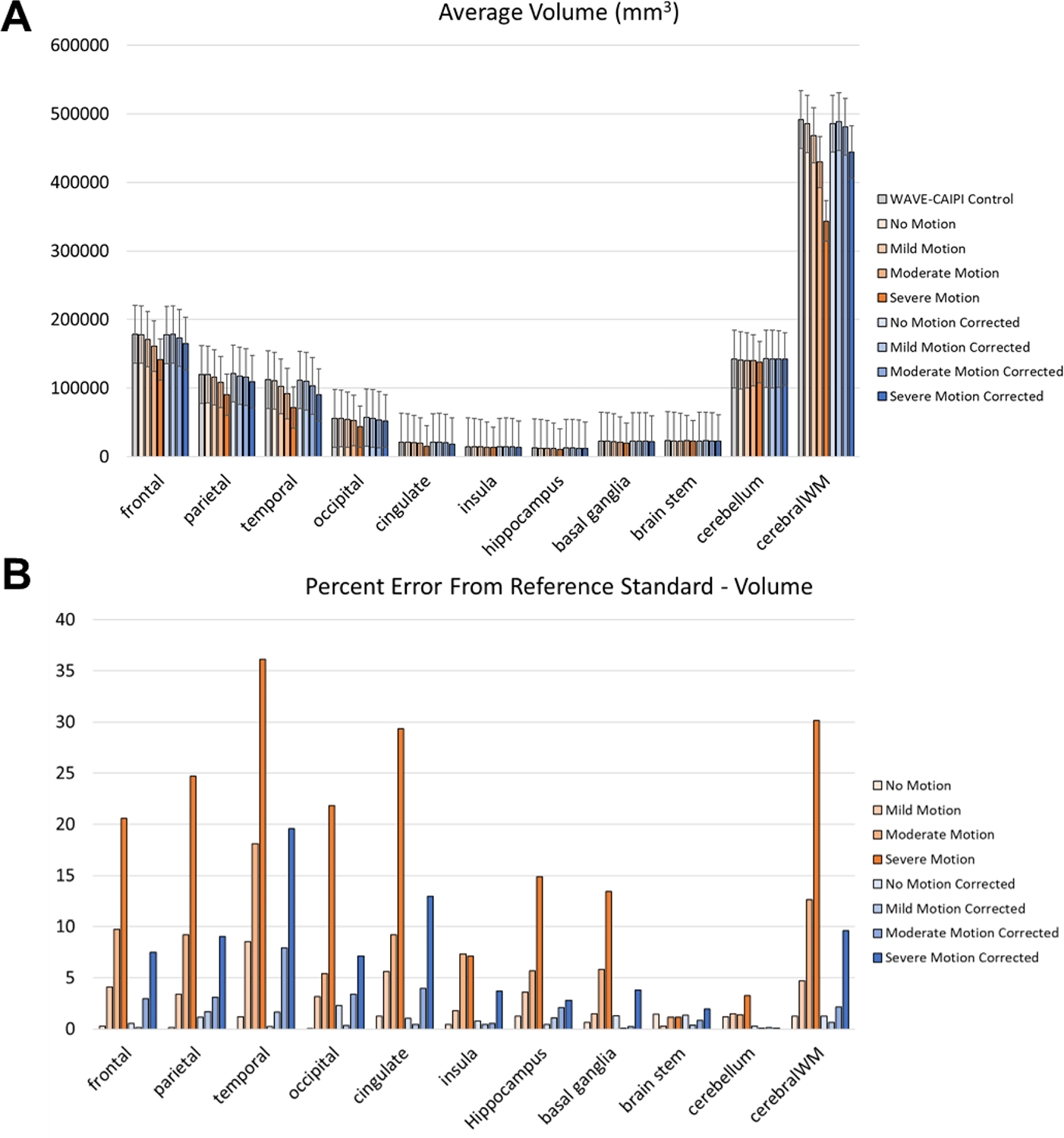
(A) Cortical volume calculations across 11 anatomical regions in the brain averaged over 12 volunteers in part (1) of our study. Increasing motion severity results in proportionately lower calculated volumes (orange bars) relative to the Wave-CAIPI control reference standard scan (gray bars). Applying motion correction with SAMER (blue bars) generally restores cortical volumes to values similar to what they were in the Wave-CAIPI scan. (B) Percent error of volume calculations for each motion state compared to the reference standard Wave-CAIPI control scan. Applying SAMER reduces the percent error of the volume calculations by up to 66 % in the cerebral white matter. The temporal lobe and cerebral white matter are the most affected by motion, and consequently see the most benefit from motion correction.

**Fig. 5.**
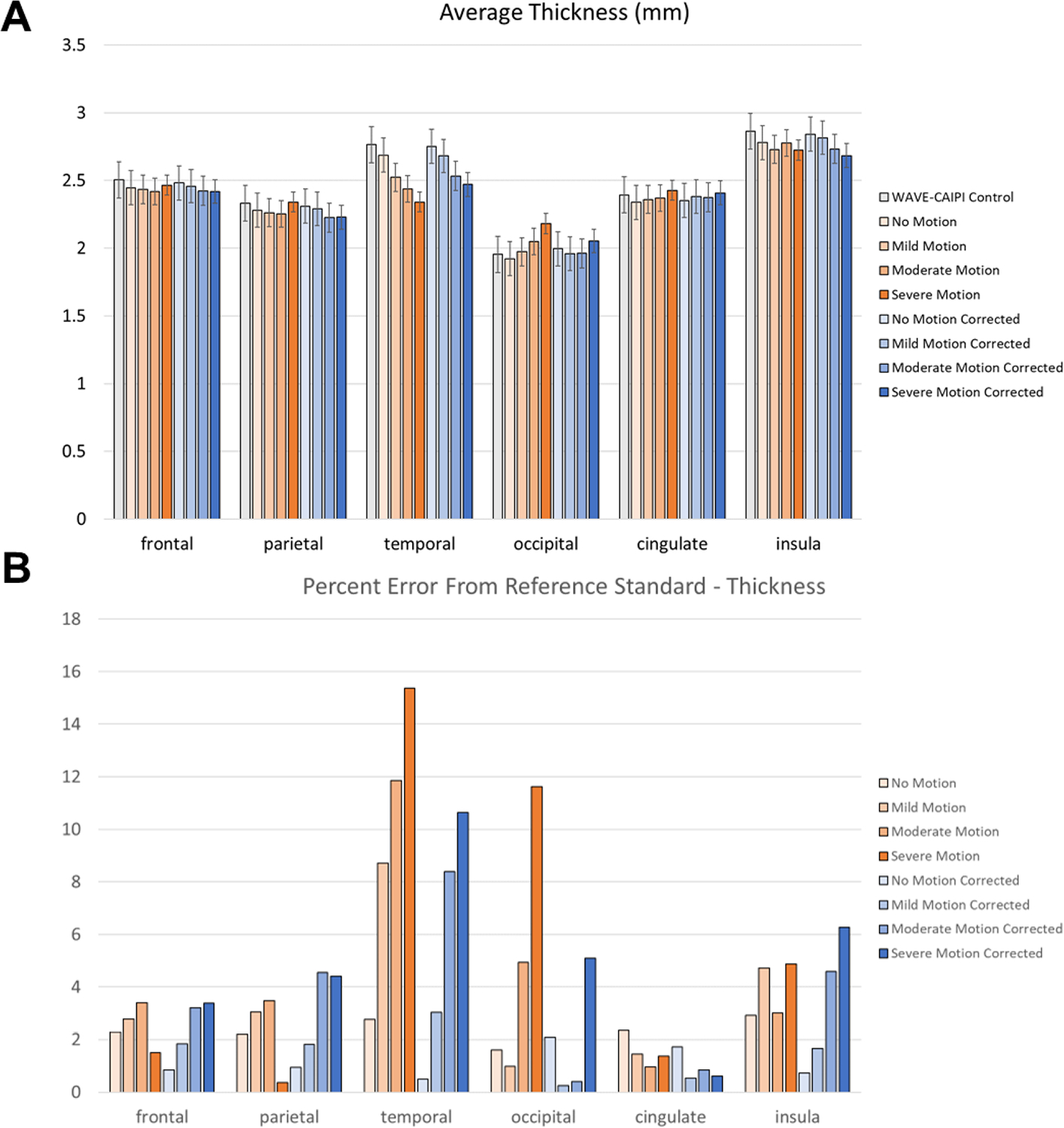
(A) Cortical thickness calculations across 6 anatomical regions in the brain averaged over the 12 volunteers. Thickness values calculated on motion-corrupted (orange bars) and SAMER-corrected (blue bars) scans are compared to the Wave-CAIPI control reference standard scan (gray bars). (B) Percent error of cortical thickness calculations for each motion state compared to the reference standard Wave-CAIPI control scan. Applying SAMER reduces the percent error of the thickness calculations by up to approximately 50 % in the case of severe motion of the occipital lobe. Overall, the benefit of motion correction on cortical thickness is less than for the cortical volume, and no significant difference is seen for several anatomical regions.

Quantitatively, errors introduced by motion artifact resulted in FreeSurfer generally underestimating the reference volume (Fig. 4A) in a manner directly proportional with the degree of motion, consistent with previously reported degradation of FreeSurfer performance in motion-degraded images (Reuter et al., 2015). The most affected anatomical areas were the temporal lobe and the cerebral white matter, with percent error values on severe motion scans reaching 36 % and 30 % compared to the reference standard scans, respectively (Fig. 4B). At the same time, SAMER considerably reduced the volume calculation error across all anatomical areas; for example, the percent error values corresponding to the temporal lobe and cerebral white matter were reduced to 20 % (relative reduction of 44 %) and 10 % (relative reduction of 66 %), respectively. Estimates of the cortical thickness behaved similarly to the ones for volume in that the temporal lobe is the most affected (Fig. 5A). However, the magnitude of the percent error is generally less across the examined anatomical areas (Fig. 5B), with the highest value for the severe motion scans being 15 % for the temporal lobe. SAMER’s contributions to improving thickness estimates were more modest. For example, the temporal lobe percent error for severe motion was reduced to 11 %. There were also neuroanatomical areas that showed no significant correction, such as the insula. Interestingly, although not statistically significant, the largest relative reduction in error with SAMER occurred in the occipital lobe (11 % to 4 % - a relative reduction of 64 %), where severe motion resulted in overestimation of the cortical thickness, in contrast to the other anatomical areas.

Statistically, SAMER results in significant improvements in calculated cortical volume between uncorrected and corrected scans in the frontal and temporal lobes and the cingulate gyrus for mild motion, in the temporal lobe, hippocampus, insula, cerebellum, and cerebral white matter for moderate motion (Table 3). However, for thickness, SAMER only statistically improved estimates in the temporal lobe for mild motion. For severe motion, although mean differences between uncorrected and corrected volumes and thicknesses are high (Figs. 4 and 5), no statistical comparisons met the significance thresholds of 0.0011 for volume and 0.0021 for thickness, with p-values for most comparisons reaching ~0.003. This was likely due to the smaller sample size that resulted from FreeSurfer failing to provide morphometric results in 6 of the 12 uncorrected severe motion cases. However, because the differences in the morphometric measures for the 6 cases where these were available for both pre- and post-correction scans are large, with a larger number of paired cases, these comparisons would likely meet the statistical significance threshold. Importantly, for individual anatomical regions there was no significant difference in cortical volume for post-correction “no motion” scans compared to their pre-correction counterparts; however, there was a small statistically significant increase in cortical thickness for post-correction “no motion” scans in the frontal, parietal, temporal, and occipital lobes.

In addition to analyzing the effect of SAMER motion correction on morphometric cortical volume and thickness measurements for different qualitative motion states, we also examined the relationship between the morphometric measures and a quantitative root mean square (RMS) displacement-based motion metric (Reuter et al., 2015). There was a generally positive correlation between the qualitative motion grades and the quantitative motion metric (Supplemental Fig. 1). However, the correlation was imperfect and there were cases where qualitatively perceived motion did not match quantitative motion metrics (Supplemental Fig. 2).

**Fig. 6.**
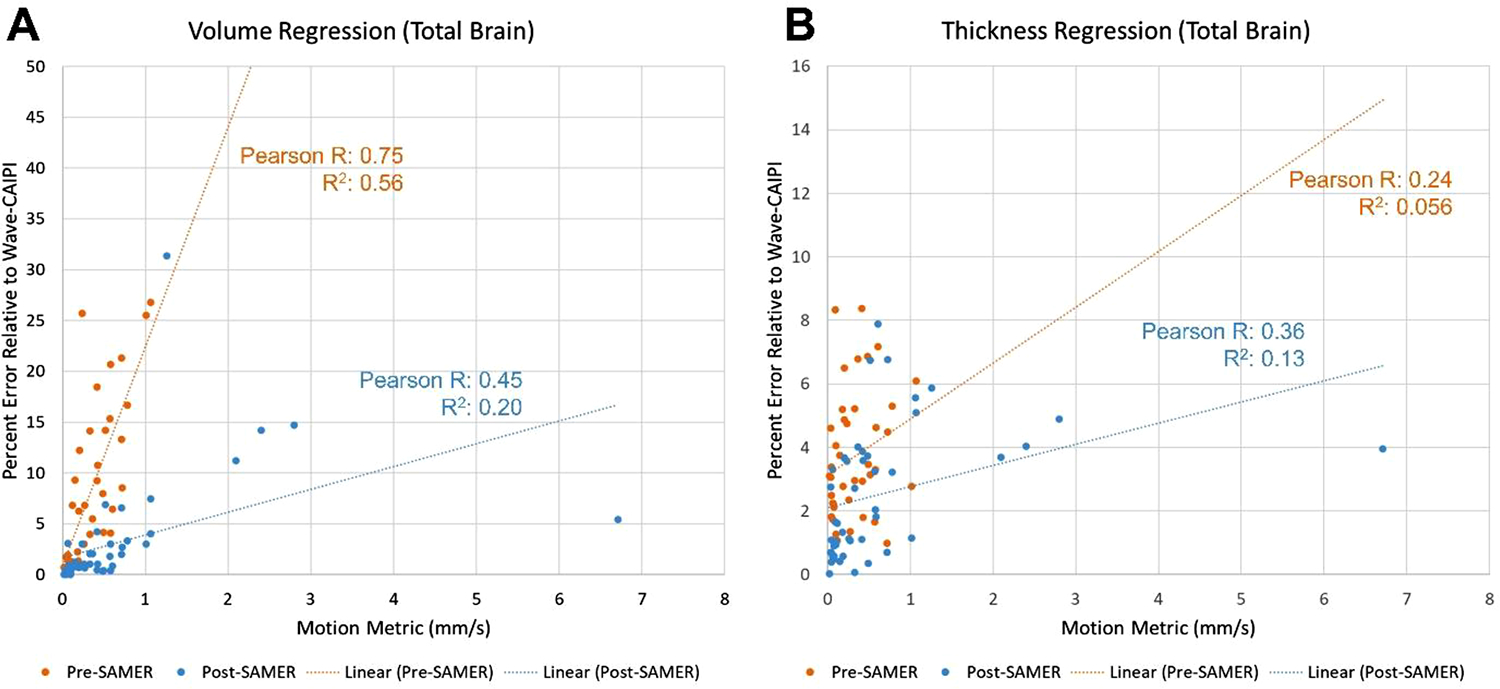
Linear regression of the percent error relative to the Wave-CAIPI reference standard scan versus quantitative motion metric values in mm/s for the cortical volume (A) and thickness (B) of the total brain, for pre-motion-correction (orange) and post-motion-correction scans in 12 volunteers. Pre-motion-correction scans for which FreeSurfer could not calculate volume or thickness are excluded. Pearson correlation coefficients and R^2^ values are displayed adjacent to each regression line. For both the cortical volume and thickness, the slope of the regression line decreases after motion correction, illustrating the reduction in percent error even at high degrees of motion. However, for cortical thickness, the Pearson correlation and R^2^ values are higher for post-motion-correction scans.

We performed linear regression analysis of the quantitative motion metric versus the percent error in cortical volume (Fig. 6A) and thickness (Fig. 6B) for the total brain. These plots demonstrate that motion correction lowers the Pearson correlation between the metric and the percent error for the volume: the Pearson correlation coefficient was 0.75 before motion correction and 0.45 after motion correction. However, the correlation values for the thickness (0.24 pre-correction and 0.36 post-correction) are persistently low. Nevertheless, the slope of the regression line decreases after motion correction for both cortical volume and thickness.

We further stratified the linear regression analysis for pre- and post-SAMER-correction scans for each of the four possible motion states for the total brain. The stratified regression analysis for volume demonstrates low Pearson coefficients for the pre-SAMER-correction scans (ranging from 0.23 – 0.32), but a wide range of Pearson correlation coefficients for the post-SAMER-correction state (ranging from 0.087 – 0.90). Nevertheless, the overall appearance of the regression lines is consistent with the trend observed for the non-stratified data (Supplemental Fig. 3). The stratified regression analysis for thickness reveals no consistent pattern in the regression lines, with Pearson correlation coefficients ranging from −0.70 – 0.14 for the pre-SAMER-correction state and 0.089 – 0.52 for the post-SAMER-correction state (Supplemental Fig. 4).

**Fig. 7.**
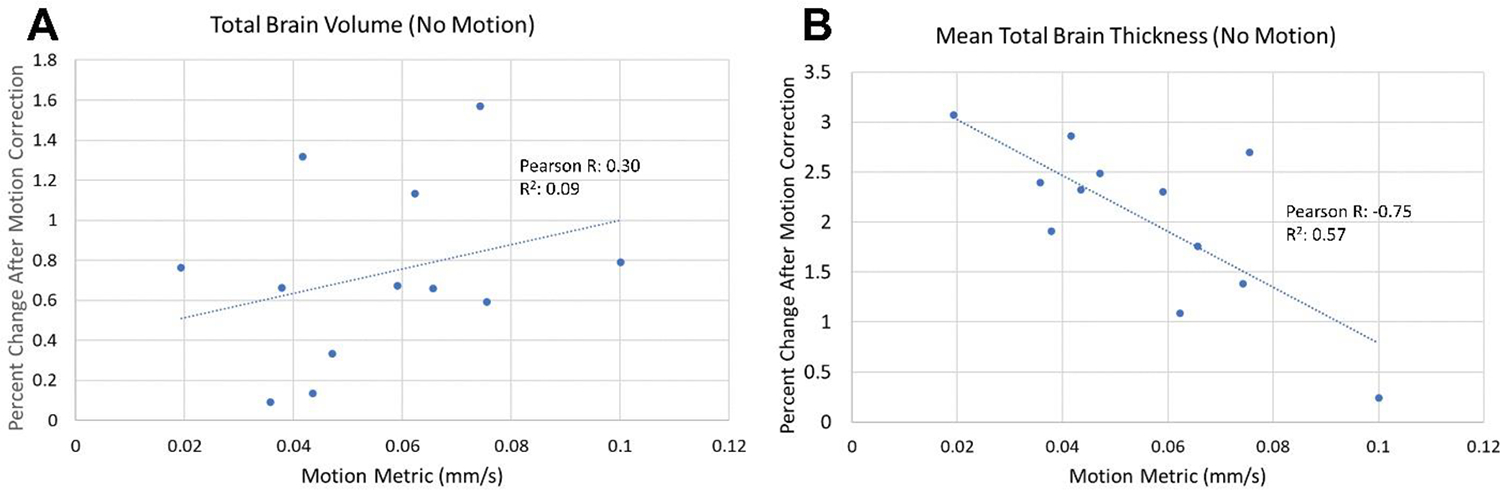
Linear regression of the absolute percent change in the total brain cortical volume (A) or thickness (B) after applying SAMER motion correction for the “no motion” scans of 12 volunteers versus motion metric values in mm/s. Pearson correlation coefficients and R^2^ values are displayed on the plots. The mean percent change after motion correction was 0.73 for volume and 2.04 for thickness. The percent change in thickness displays a negative correlation with the motion metric.

The Pearson correlation coefficient value of −0.70 in Supplemental Fig. 4 corresponded to the “no motion” state, which prompted further analysis of the effects of correcting residual motion on cortical volume and thickness measurements for the total brain. The mean absolute percent change after motion correction in the “no motion” cases was ~0.7 % for the volume and ~2 % for the thickness. These absolute percent changes are statistically significantly greater than 0 using a one-tailed Wilcoxon test (*p* = 0.001 for volume and *p* < 0.001 for thickness). Linear regression of the percent changes in volume (Fig. 7A) and thickness (Fig. 7B) demonstrated that there is a positive correlation between the motion metric and the percent change in volume (Pearson correlation: 0.30) and a negative correlation between the motion metric and the percent change in thickness (Pearson correlation: −0.75).

### 3.2. Part (2): Effect of SAMER motion correction on estimated cortical volume and cortical thickness in patients undergoing evaluation for memory loss

Twenty-nine patients (mean age 62.4 ± 14, 11 women; Table 1) underwent memory loss protocol brain MRI on a single outpatient MRI scanner. All SAMER-MPRAGE scans demonstrated at least “minimal” motion corruption, as defined by a previously validated 5-point motion corruption scale (Lang et al., 2023), with larger numbers indicating greater degrees of motion. The motion artifact we observed in uncorrected SAMER-MPRAGE scans across patients demonstrated a mean score of 2.45 (Supplemental Table 2), which would be classified as between “minimal” and “mild”. Wave-CAIPI MPRAGE scans displayed less apparent motion artifact, with a mean score of 1.89 (Supplemental Table 2), a statistically significant apparent difference (*p* < 0.01, two-tailed paired Student’s *t*-test). However, the motion score of 1.89 still corresponded to “minimal” motion, indicating that, on average, patients still moved during their Wave-CAIPI MPRAGE acquisitions. Almost all patients exhibited either “minimal” or “mild” motion artifact in their SAMER pre-correction scans (Supplemental Table 3).

**Fig. 8.**
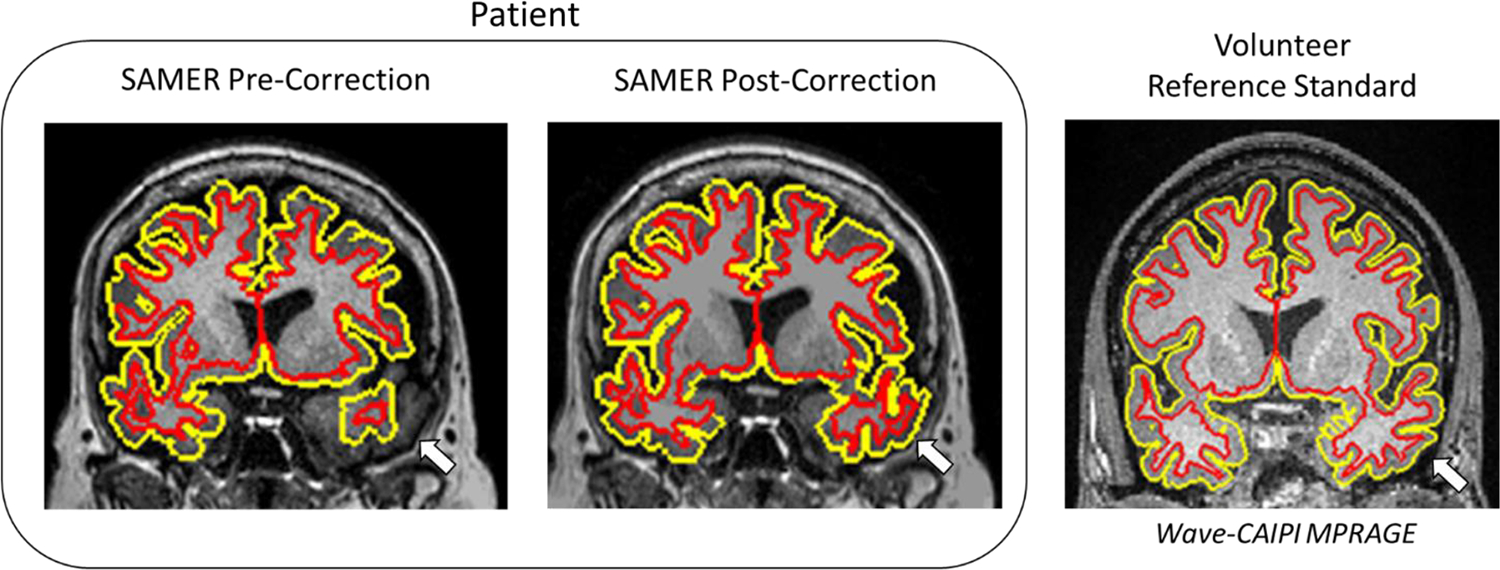
Representative FreeSurfer segmentation on a coronal slice demonstrating the inner and outer cortical surface on motion corrupted and corrected scans for one patient from part (2). Segmentation of the left temporal lobe is especially improved after motion correction in this patient (arrow), resembling that of a volunteer reference standard.

FreeSurfer cortical volume and thickness calculations were successfully completed for all reconstructed images. Applying SAMER to the patients’ motion-corrupted scans resulted in qualitatively improved cortical segmentations (Fig. 8), that resembled the trends seen in the healthy volunteer study reported in Part (1) of this work. Based on the results from Part (1), we hypothesized that calculated cortical volume and thickness values would increase after successfully applying motion correction on motion-corrupted scans.

**Fig. 9.**
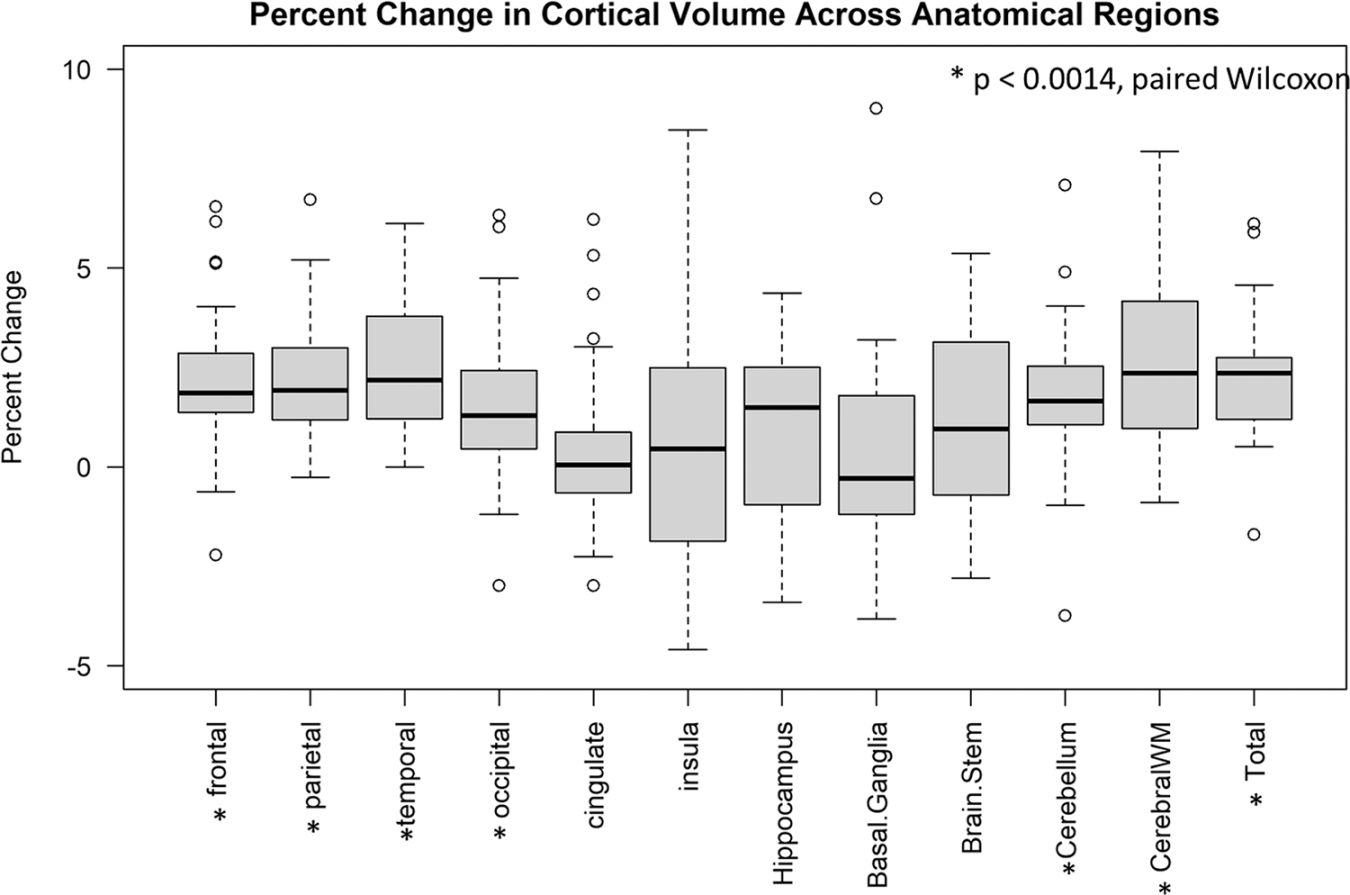
Boxplot of percent change in cortical volume (post motion correction – pre motion correction) by region for the 29 patients in part (2) of our study. Statistical significance was assessed by paired Wilcoxon rank sum tests, with a Bonferroni-corrected threshold of *p* < 0.0014.

We observed statistically significant increases in calculated volumes after applying SAMER that were most pronounced in the temporal, frontal, and parietal lobes, followed by the cerebellum and cerebral white matter (Fig. 9 and Table 4). However, the magnitude of the effect was generally small (mean of ~2 %, reaching up to ~4 % for the temporal lobe), likely because the motion artifact we observed in patients was between “minimal” and “mild” to begin with – this is in accordance with the results we observed in volunteers form part (1), where, across anatomical regions, the average increase in calculated cortical volumes following correction of “mild” motion was ~3 %. When comparing SAMER-MPRAGE pre- and post-correction scans to Wave-CAIPI MPRAGE, cortical volume calculations demonstrate a reduction in percent error for the frontal, parietal, temporal, and occipital lobes (Supplemental Fig. 5), with the largest relative reduction being from ~4 % to ~0.5 % in the parietal lobe. On the other hand, percent error slightly increased after SAMER correction for the hippocampus and basal ganglia (for example, from ~1 % to ~5 % in the hippocampus). This was due to Wave-CAIPI MPRAGE systematically underestimating the cortical volumes for the hippocampus and basal ganglia in the patients, such that their volumes are lower than those calculated from the pre-correction SAMER MPRAGE scans (Supplemental Fig. 6).

**Fig. 10.**
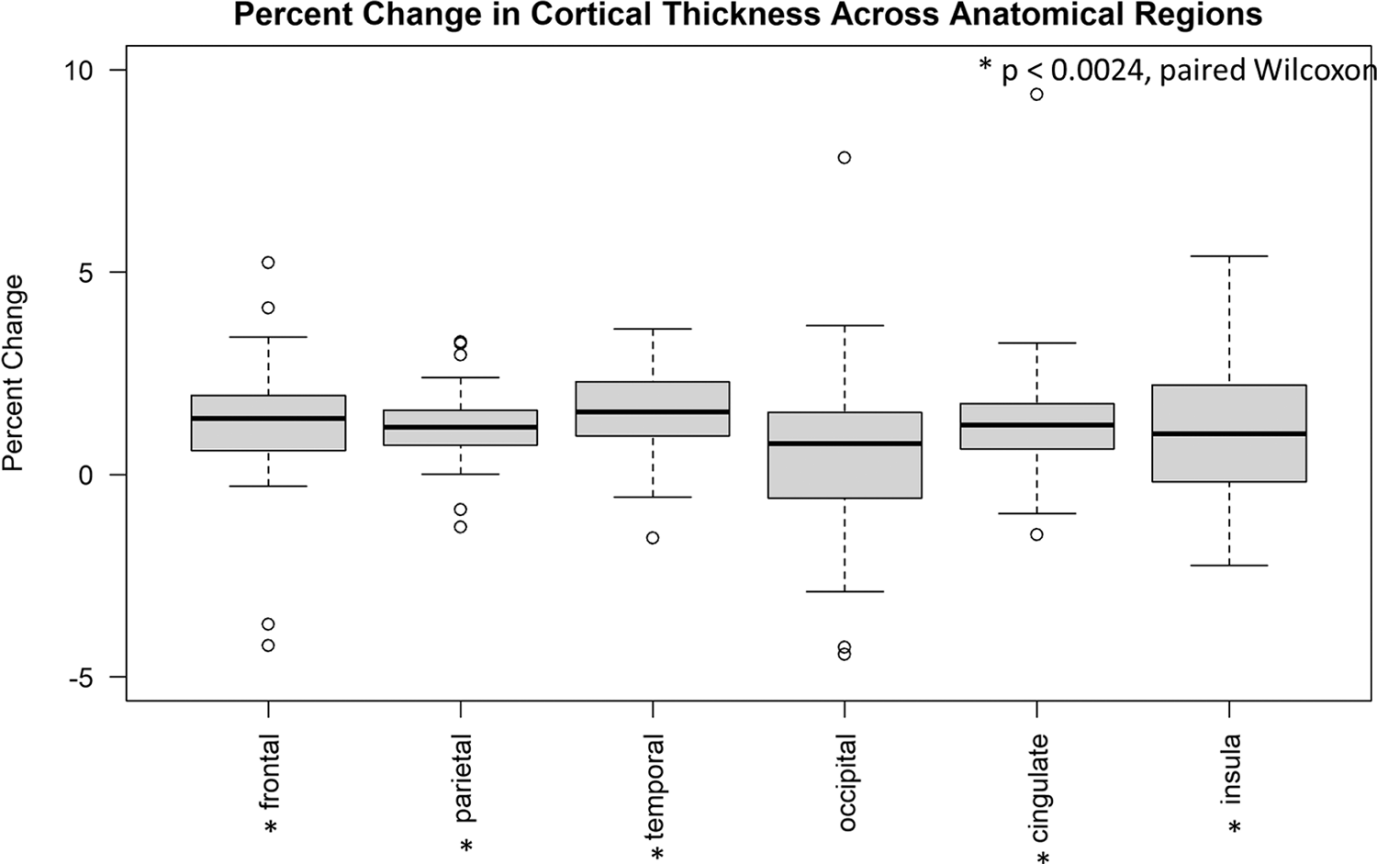
Boxplot of percent difference in cortical thickness (post motion correction – pre motion correction) by region for the 29 patients in part (2) of our study. Statistical significance was assessed by paired Wilcoxon rank sum tests, with a Bonferroni-corrected threshold of *p* < 0.0024.

Cortical thickness measurements also demonstrated increases across anatomical regions, with statistically significant results observed for the temporal, frontal, and parietal lobes and the cingulate gyrus, as well as the total brain (Fig. 10 and Table 4). Like for the cortical volumes, effect sizes were small (mean of ~1 %, reaching up to ~1.4 % for the temporal lobe) but were in accordance with our results on volunteers with “mild” motion, where the average increase in calculated cortical thickness following motion correction was ~2 %. When comparing SAMER-MPRAGE pre- and post-correction scans to Wave-CAIPI MPRAGE, cortical thickness calculations demonstrated a reduction in percent error for the frontal, parietal, and temporal lobes, as well as the total brain (Supplemental Fig. 7). The magnitudes of decrease in percent error were similar to those seen in the cortical volume calculations; for example, the total brain volume decreased from ~3 % to ~1 %.

Given that subgroups of patients exhibited different motion states (Supplemental Table 3), we also examined whether there were any differences in distributions of cortical volume or thickness between these subgroups. Out of the 29 patients, 17 exhibited “minimal” motion, 11 exhibited “mild” motion, and 1 exhibited “moderate” motion. Because of this limited distribution, we dichotomized our evaluation into either an “at least mild” (mild+) motion group or an “at most minimal” (min−) motion group. Ultimately, there were no significant differences across anatomical in the distributions of cortical volume (Supplemental Fig. 8) or thickness (Supplemental Fig. 9) across anatomical regions for these motion states.

## 4. Discussion

In this work, we used data from patients and healthy volunteers to characterize quantitatively how SAMER addresses motion corruption in 3D MPRAGE sequences. Motion is a major barrier limiting the utility of volumetric brain MRI for clinical research, particularly in patients undergoing evaluation for memory loss, who may be more motion prone than the typical healthy young adult population. Our work is novel because 1) it provides the first systematic characterization of the effect of SAMER on brain morphometry and 2) provides an application of SAMER in a realistic clinical population. For this purpose, we used FreeSurfer, an established image analysis package, which is necessary to assess the robustness of motion correction. Given that our study design obtains multiple scans the same subjects, we used the longitudinal FreeSurfer pipeline to limit the errors in volumetric evaluations by reducing within-subject variability, as in our previous work (Longo et al., 2020). This reduced variance between timepoints/datasets from longitudinal analysis leads to an underestimation of effect sizes, so our results reflect a more conservative estimation of the improvement in morphometric measures gained from applying SAMER.

In the healthy volunteers studied in part (1), greater degrees of motion corruption resulted in proportionate decreases in the measured values of cortical volume and thickness across most anatomical regions in the brain. Applying motion correction with SAMER increased the estimated cortical volume and thickness, restoring these volumetric measurements to their non-motion-corrupted values and to those observed in the Wave-CAIPI-MPRAGE “reference standard” across qualitatively rated motion states. Linear regression analysis of the percent error in cortical volume and thickness against a quantitative motion metric for the total brain overall corroborated these results by demonstrating a reduction in each unit of percent error gained for each unit of motion metric, although the linear relationship for the cortical thickness was weaker. For the cortical volume, we observed the same trends when stratifying the regressions by individual motion states. However, we observed no consistent trend for the thickness when stratifying by qualitative motion state, likely due to a combination of the weaker baseline effect and the reduction in available data caused by subgrouping.

There was an imperfect correlation between motion amplitude and qualitative motion ratings. These variations may be due to differences in the character of random motion across volunteers as well as complex dependencies of motion artifact on the position of data within k-space. A systematic evaluation of the effects of specific motion types on artifacts is a subject for future research.

Importantly, our volunteer study included a control in which SAMER was applied to “no motion” scans. The results of FreeSurfer segmentation on the SAMER “no motion” scans before and after motion correction showed no significant change in cortical volume values for any individual anatomical region. Therefore, any measured increase in cortical volume after applying SAMER was attributed to successful motion correction and not any confounding characteristics intrinsic to the novel sequence reordering or motion correction algorithm, in keeping with previous findings (Polak et al., 2022). However, statistically significant increases in cortical thickness measurements were observed after motion correction even in the “no motion” case, which may plausibly represent the effect of correction of very small amounts of subject motion in these cases. Alternatively, this may be due to the introduction of a small amount of systematic error by the SAMER algorithm into the estimation of cortical thickness by FreeSurfer, for example, by falsely correcting motion when it is not present. Our analysis suggests that the mean percent change in volume is 0.7 % and the mean percent change in thickness is approximately 2 % across the total brain for “no motion” cases. Given we observed these results in a relatively small population of 12 subjects, the statistically significant ~2 % difference in cortical thickness will be important to consider when studying larger populations in clinical or neuroscience cohort studies because it suggests there can be variability in repeated thickness measurements obtained in the same subject. In addition, we observed a slight negative correlation of the percent change in thickness with the quantitative motion metric. The negative correlation observed for the thickness suggests that rather than SAMER correcting for residual motion, there is another source of systematic bias, which could be explored in a future study.

In part (2) of our study, we studied the impact of motion correction in the real-life clinical use case of cortical volume and thickness determination in patients undergoing evaluation for dementia. We found statistically significant increases in measured cortical volumes and thicknesses across anatomical regions, demonstrating that SAMER motion correction could be applied successfully to patient data for quantitative volumetric calculation and that the post-correction volumes and thicknesses might be more accurate representations of their “true” values. This is supported by the values of cortical volume and thickness obtained from standard Wave-CAIPI MPRAGE scans being generally closer to those obtained from post-correction SAMER-MPRAGE scans compared to the pre-correction scans. While for the patients we did not see significant differences in cortical volume based on the degree of motion artifact that was corrected, by and large only “minimal” or “mild” motion states were represented, which caused limited motion artifact to begin with and may have obscured any detectable differences.

In both volunteers and patients, the anatomical areas whose volume and thickness measurements were most affected by motion artifact and benefitted the most from SAMER motion correction tended to be more peripherally located in the brain: the cortical surfaces of the frontal, parietal, and temporal lobes stand out. In addition, the cerebral white matter also especially benefited from motion correction, which may be due to large portions of the white matter being located more peripherally in the brain. In contrast, the deep brain structures like the hippocampus and basal ganglia were overall less affected by motion artifact in patients and volunteers. That the patterns of the effect of motion artifact and correction held across volunteers and patients lends additional support to aid in the interpretation of the results we obtained in patients.

An interesting observation in our clinical dataset was that applying SAMER increased the volume percent error relative to Wave-CAIPI MPRAGE scans due to a systematic underestimation of hippocampal and basal ganglia volume. We did not observe this in the volunteers. This may have been due to the Wave-CAIPI MPRAGE scans not being specifically controlled for motion in the patients; both the presence of motion and the differing motion types from the volunteers may have contributed to differences in results. Furthermore, in our clinical dataset, the reference standard Wave-CAIPI MPRAGE scans visually appeared to have less motion artifact than the SAMER-MPRAGE pre-correction scans. This observation may reflect less motion overall during the Wave-CAIPI MPRAGE scans due earlier ordering in the acquisition (all the Wave-CAIPI MPRAGE scans were performed first and the research SAMER MPRAGE scans were done last), in addition to the intrinsic robustness of Wave-CAIPI MPRAGE to motion (Oh et al., 2023).

Based on our results, an example of how to apply SAMER for the clinical evaluation of cortical volume would be to consider how this value would be affected on a specific anatomical region following correction in a patient with motion-corrupted images. If the volume increases after correction, then it suggests that motion artifact was corrected, and the patient is unlikely to have a decreased volume in the anatomical region in question. On the other hand, if the volume does not change after applying SAMER, then this suggests that the volume of the anatomical region of interest is truly decreased. Future work may also compare SAMER to other motion correction approaches, such as deep-learning-based methods (Nalini et al., 2023), in the setting of assessing volumetric calculation accuracy.

An important consideration in our study design was whether the conclusions drawn from the healthy volunteer study in part (1) are directly applicable to the dementia-workup patients in part (2). Although both the volunteers and patients were predominantly male (8/12 volunteers and 18/29 patients), the volunteers tend to be younger than the patients. The generalizability of the findings is also limited by the small sample sizes of both studies. Nevertheless, we observed that applying motion correction to the dementia-workup patients’ mildly motion-corrupted data resulted in higher calculated volumes across anatomical regions, which was consistent with the findings in the volunteer data sampled across a wider range of motion. In addition, we also found that the magnitudes of the volume increases were similar between the patients and the “mild” motion-corrupted scans of the volunteers. The motion artifact observed in the patient dataset was overall mild, leading to limited room for improvement with motion correction. Nevertheless, we observed small, though statistically significant, effect sizes in the patient cohort, which we interpreted as a consistent and important translation of the findings from the healthy volunteer study. These findings merit validation in patients with more severe motion, which were not represented in the patients scanned in this outpatient setting. A follow-up study on a larger clinical population with more severe dementia is important to validate the findings of our study, and efforts are underway to address this question as a separate work.

We emphasize that the clinical evaluation of motion correction algorithms is difficult to implement and apply in the clinical setting due to numerous practical hurdles, including limits on scanner time, adequate training of MRI technologists, and manual data collection for research sequences like SAMER. Some studies resort to using simulated motion artifacts (Al-Masni et al., 2022), but the clinical translation of conclusions from such studies remains uncertain and ultimately requires experimental validation. Furthermore, the benchmarking of approaches to motion correction has largely focused on how it affects the subjective grading of image quality, and few studies have quantitatively analyzed the effects of motion correction on volumetric measurements in a practical clinical setting. One prior study (Sarlls et al., 2018) analyzed prospective motion correction and subsequent volumetric measurements when applied to a specific type of motion (a figure-eight trajectory of the nose). Prior work has also demonstrated a relationship between increasing motion severity and decreased cortical volume and thickness measurements (Reuter et al., 2015). Another study examined the effect of a prospective motion correction system on gray matter volume in healthy adult volunteers undergoing different types of qualitative motion, including “nodding”, “shaking”, and “moving freely” (Tisdall et al., 2016), finding that a quantitative motion metric had different correlations for each motion type with and without motion correction; non-motion corrected scans exhibited a negative correlation between volume and a quantitative motion metric, while motion-corrected scans exhibited a weaker negative correlation. A study on a pediatric population also demonstrated that motion-corrected cortical volumes were higher than non-motion-corrected volumes (Kecskemeti et al., 2021). Our study supports the findings of this previous work by being the first to systematically illustrate the effects of SAMER, a recently developed, mechanistically different motion correction approach, on quantitative brain morphometry. In addition, this work provides an application of SAMER in a realistic clinical population of patients undergoing evaluation for memory loss, distinguishing it from prior work in motion correction for brain MRI, which has mostly used healthy volunteers as subjects. Our work ultimately represents an important step in the clinical translation of SAMER. More broadly, we consider this work to represent an important step toward resolving motion artifact in cortical volumetric analysis, which is a practical and ubiquitous problem that will continue to attract attention as the volume of patients undergoing MRI evaluation for memory loss grows with the increasing availability of Alzheimer’s disease therapies (Cogswell et al., 2022).

## 5. Conclusion

The SAMER technique improves severe motion artifacts and can potentially turn non-diagnostic-quality scans into ones that allow for accurate computation of cortical volume and thickness. Our results suggest that SAMER has strong potential for use in practical clinical settings and can contribute to diagnosing and managing Alzheimer’s disease and other neurodegenerative disorders.

## Supplementary Material

1

## Figures and Tables

**Table 1:** Demographic characteristics for the 12 volunteer subjects studied in Part (1) and 29 clinical subjects studied in Part (2) of this work. Part (1) aimed to characterize the effect of SAMER on varying degrees of motion in a controlled experimental setting, while Part (2) aimed to apply SAMER in a clinical setting on outpatients being evaluated for dementia. Clinical indications for brain MR imaging are listed for Part (2).

Part (1) - Volunteer Study	
	Value
No. of subjects	12
Mean age (years ± SD)	48.3 ± 13
Sex (F:M)	4:8
Part (2) - Clinical Study	
	Value
No. of subjects	29
Mean age (years ± SD)	62.4 ± 14
Sex (F:M)	11:18
Clinical indication for MR imaging	N (%)
*Memory loss*	11 (38 %)
*Dementia*	4 (13.7 %)
*Headache*	4 (13.7 %)
*Neurologic deficit*	3 (10.3 %)
*Traumatic Brain Injury (TBI)*	1 (3 %)
*Parkinson disease*	1 (3 %)
*Schizophrenia*	1 (3 %)
*Other*	4 (13.7 %)

**Table 2:** Acquisition parameters for SAMER and Wave-CAIPI MPRAGE sequences, demonstrating that they are nearly identical. The only difference is that the echo time (TE) of SAMER-MPRAGE was 3.3 ms, compared to 3.2 ms for Wave-CAIPI MPRAGE.

Acquisition Parameter	SAMER-MPRAGE	Wave-CAIPI-MPRAGE
FOV (mm)	256×256×192	256×256×192
Matrix (mm)	256×256×192	256×256×192
TR/TE/TI (ms)	2300/3.3/1000	2300/3.2/1000
Flip Angle (degrees)	8	8
Bandwidth (Hx/Pixel)	200	200
Acquisition Time (min)	2:39	2:39

**Table 3:** Median cortical volume (mm^3^) and thickness (mm) values for the 12 volunteers in part (1) for each of four motion states: none, mild, moderate, and severe, both before and after applying motion correction. In addition, paired Wilcoxon signed rank test P values for volume and thickness comparisons are listed. Statistical significance based on Bonferroni-corrected thresholds for statistical significance of <0.0011 for cortical volume and <0.0021 for cortical thickness is noted with an asterisk (*). Interquartile ranges are listed in brackets. Because statistical comparisons for severe motion cases were based on the 6 volunteers from whom morphometric measures were able to be calculated before motion correction, their p-values are listed as “>0.0011″ or “>0.0021″.

	Motion Grades and Corresponding SAMER Correction P Values											
	Cortical Volume (mm^3^) [IQR]											
Anatomical Region	None	None Corrected	None P Value	Mild	Mild Corrected	Mild P Value	Moderate	Moderate Corrected	Moderate P Value	Severe	Severe Corrected	Severe P Value
frontal	177,923 [19,954]	177,405 [18,862]	0.791	171,102 [28,240]	178,188 [19,605]	0.0005*	161,023 [36,818]	173,129 [15,823]	0.002	141,709 [41,645]	165,039 [30,506]	>0.0011
parietal	119,737 [16,471]	121,216 [15,674]	0.110	115,811 [17,348]	117,836 [17,728]	0.064	108,821 [25,400]	116,160 [15,487]	0.007	90,279 [22,669]	109,028 [19,495]	>0.0011
temporal	110,683 [16,141]	111,748 [16,077]	0.027	102,461 [21,042]	110,156 [17,275]	0.0005*	91,741 [26,161]	103,123 [16,668]	0.001*	71,534 [16,890]	90,061 [16,460]	>0.0011
occipital	55,790 [14,413]	57,077 [14,315]	0.005	54,031 [14,015]	55,611 [13,091]	0.092	52,789 [13,115]	53,895 [16,271]	0.577	43,623 [18,282]	51,820 [15,419]	>0.0011
cingulate	20,999 [4837]	21,034 [4437]	0.301	20,061 [5238]	21,163 [4485]	0.0005*	19,302 [5575]	20,417 [3976]	0.005	15,021 [5937]	18,501 [1946]	>0.0011
insula	14,370 [2514]	14,320 [1919]	0.622	14,172 [1732]	14,366 [2294]	0.519	13,375 [2592]	14,356 [2391]	0.001*	13,407 [3733]	13,895 [2482]	>0.0011
hippocampus	12,487 [2173]	12,588 [2146]	0.042	12,188 [2178]	12,506 [2107]	0.007	11,922 [1999]	12,375 [2362]	0.001*	10,758 [2681]	12,286 [1957]	>0.0011
basal ganglia	22,575 [3191]	22,721 [3759]	0.204	22,100 [3319]	22,413 [3975]	0.064	21,131 [4005]	22,386 [3877]	0.014	19,418 [5370]	21,579 [3268]	>0.0011
brain stem	22,748 [5701]	22,772 [7557]	0.129	23,026 [5810]	23,174 [5576]	0.470	23,347 [4853]	22,894 [6858]	0.831	22,825 [6058]	22,639 [6316]	>0.0011
cerebellum	140,753 [11,742]	142,870 [13,515]	0.016	140,354 [16,079]	142,304 [15,179]	0.002	140,467 [7636]	142,608 [14,647]	0.001*	137,821 [12,000]	142,551 [17,487]	>0.0011
cerebralWM	485,547 [89,553]	485,600 [79,609]	0.791	468,655 [97,739]	488,605 [82,683]	0.012	429,620 [137,848]	481,088 [114,580]	0.001*	343,580 [145,554]	444,416 [124,405]	>0.0011
	Cortical Thickness (mm) [IQR]											
frontal	2.45 [0.22]	2.48 [0.23]	0.0005*	2.43 [0.26]	2.46 [0.23]	0.233	2.42 [0.16]	2.42 [0.29]	0.898	2.47 [0.10]	2.42 [0.19]	>0.0021
parietal	2.28 [0.25]	2.31 [0.22]	0.0005*	2.26 [0.19]	2.29 [0.20]	0.108	2.25 [0.17]	2.23 [0.29]	0.831	2.34 [0.25]	2.23 [0.26]	>0.0021
temporal	2.69 [0.23]	2.75 [0.23]	0.001*	2.52 [0.17]	2.68 [0.21]	0.0005*	2.44 [0.15]	2.53 [0.29]	0.005	2.34 [0.30]	2.47 [0.24]	>0.0021
occipital	1.92 [0.10]	2.00 [0.16]	0.001*	1.97 [0.17]	1.96 [0.16]	0.470	2.05 [0.08]	1.96 [0.14]	0.024	2.18 [0.09]	2.05 [0.22]	>0.0021
cingulate	2.34 [0.15]	2.35 [0.18]	0.042	2.36 [0.20]	2.38 [0.14]	0.077	2.37 [0.13]	2.37 [0.17]	0.520	2.43 [0.13]	2.41 [0.19]	>0.0021
insula	2.78 [0.16]	2.84 [0.19]	0.021	2.73 [0.31]	2.82 [0.20]	0.052	2.78 [0.16]	2.73 [0.32]	0.625	2.72 [0.16]	2.68 [0.30]	>0.0021

**Table 4:** Median cortical volume and thickness values for the 29 clinical subjects in part (2) of our study, with interquartile ranges in brackets. Calculations were performed for both pre- and post-SAMER scans, as well as for WAVE-MPRAGE scans. Paired Wilcoxon signed rank test P values are listed to compare pre- and post-corrected scans to each other and to WAVE-MPRAGE. Statistical significance is based on Bonferroni-corrected thresholds for statistical significance of < 0.0014 for cortical volume and < 0.0024 for cortical thickness and noted with an asterisk (*).

Cortical Volume (mm^3^) [IQR]				Paired Wilcoxon P Values		
Anatomical Region	Pre-Correction	Post-Correction	Wave-CAIPI MPRAGE	Post-Correction vs. Pre-Correction	Post-Correction vs. Wave-CAIPI MPRAGE	Pre-Correction vs. Wave-CAIPI MPRAGE
frontal	147,056 [29,015]	150,039 [30,843]	151,096 [23,976]	0.000124*	0.722	0.000342*
parietal	95,848 [13,266]	99,065 [13,347]	99,573 [15,402]	8.84E-06*	0.722	0.000274*
temporal	92,239 [21,888]	94,856 [20,623]	99,788 [20,284]	4.00E-06*	0.231	7.45E-09*
occipital	44,698 [9164]	45,293 [8217]	45,967 [8625]	0.000156*	0.994	0.0136
cingulate	18,055 [4687]	18,146 [4763]	18,086 [4794]	0.782	0.823	0.274
insula	12,975 [3410]	12,978 [2679]	13,160 [3482]	0.375	0.500	6.32E-05*
Hippocampus	10,281 [2387]	10,711 [2554]	10,211 [2243]	0.012943167	0.541690398	0.0735
Basal.Ganglia	20,281 [4894]	20,850 [3305]	19,924 [3645]	1	0.490	0.164
Brain.Stem	20,276 [5880]	20,380 [5082]	20,658 [4384]	0.0216	0.490	1.54E-06*
Cerebellum	130,249 [26,054]	131,979 [29,159]	129,878 [25,884]	3.18E-05*	0.818	6.56E-07*
CerebralWM	437,265 [102,686]	439,823 [104,091]	442,772 [93,692]	9.31E-08*	0.868	0.00223
Total	1,024,842 [199,126]	1,047,155 [206,200]	1,055,242 [193,676]	1.60E-07*	0.782	7.45E-09*
Cortical Thickness (mm) [IQR]				Paired Wilcoxon P Values		
frontal	2.36 [0.17]	2.39 [0.17]	2.42 [0.15]	0.000451*	0.00244	0.000716*
parietal	2.13 [0.13]	2.16 [0.15]	2.21 [0.15]	5.52E-06*	1.02E-06*	3.20E-07*
temporal	2.54 [0.12]	2.57 [0.14]	2.67 [0.19]	1.67E-06*	1.04E-07*	1.49E-08*
occipital	1.86 [0.12]	1.85 [0.13]	1.89 [0.11]	0.213	0.000647*	0.000792*
cingulate	2.33 [0.15]	2.34 [0.16]	2.35 [0.13]	0.000332*	0.867	0.000792*
insula	2.79 [0.31]	2.84 [0.23]	2.82 [0.23]	0.0229	0.227	0.274
Total	2.31 [0.14]	2.35 [0.13]	2.38 [0.14]	1.60E-05*	1.54E-06*	3.20E-07*

## Data Availability

Data will be made available on request.

## Data and Code Availability Statement

All data were acquired as part of Institutional Review Board (IRB)-approved protocols at the Massachusetts General Hospital and will be made available upon reasonable request. All third-party code used in this work is available online and cited within this work.
