# Supplementary material for: Quantitative evaluation of Scout Accelerated Motion Estimation and Reduction (SAMER) MPRAGE for morphometric analysis of brain tissue in patients undergoing evaluation for memory loss
Source: Neuroimage. Author manuscript; Available in PMC 2024 Oct 23. (PMC11498920; doi:10.1016/j.neuroimage.2024.120865)

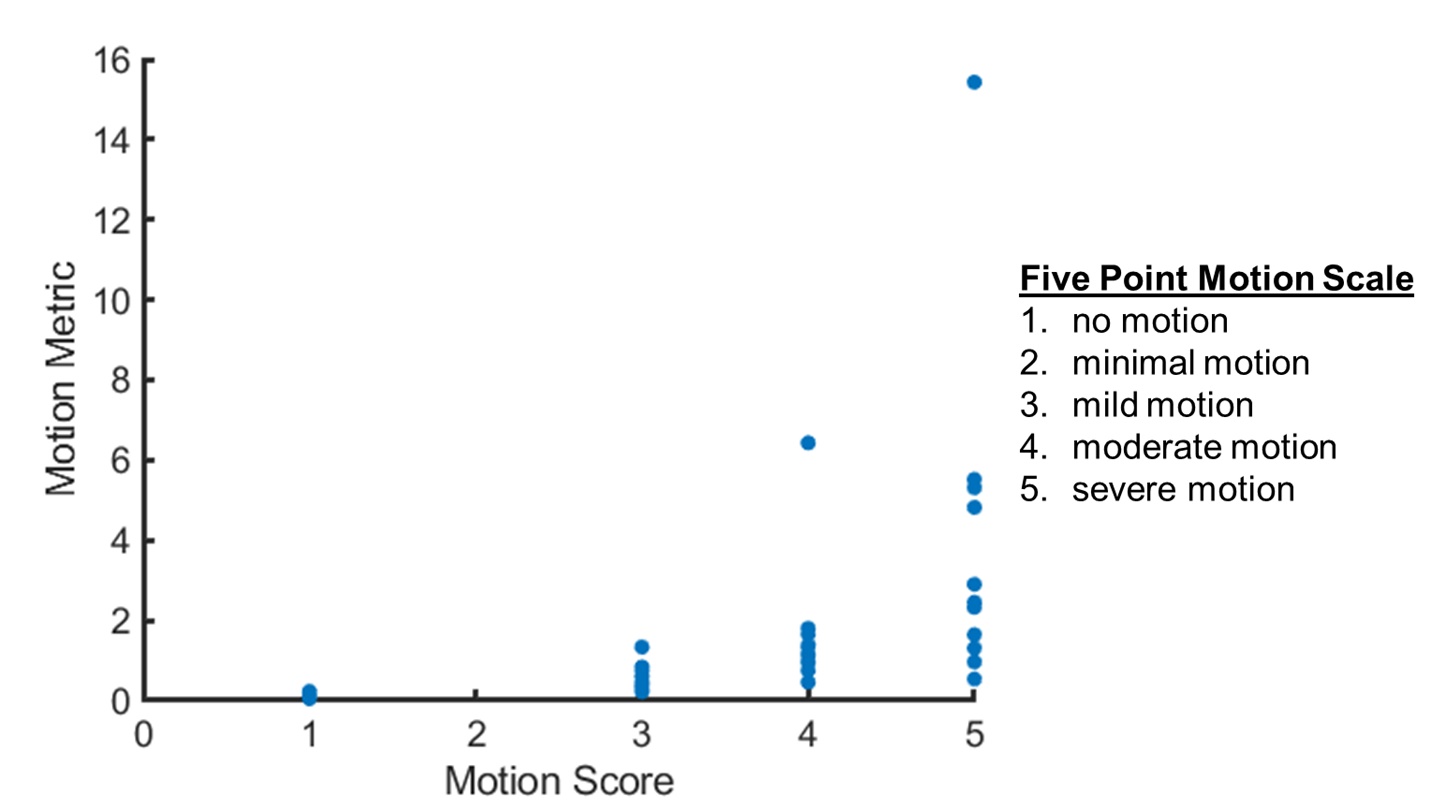


**Supplemental Figure 1.** Correlation between quantitative motion metric and corresponding qualitative motion ratings in the 12 volunteers in part (1) of our study.


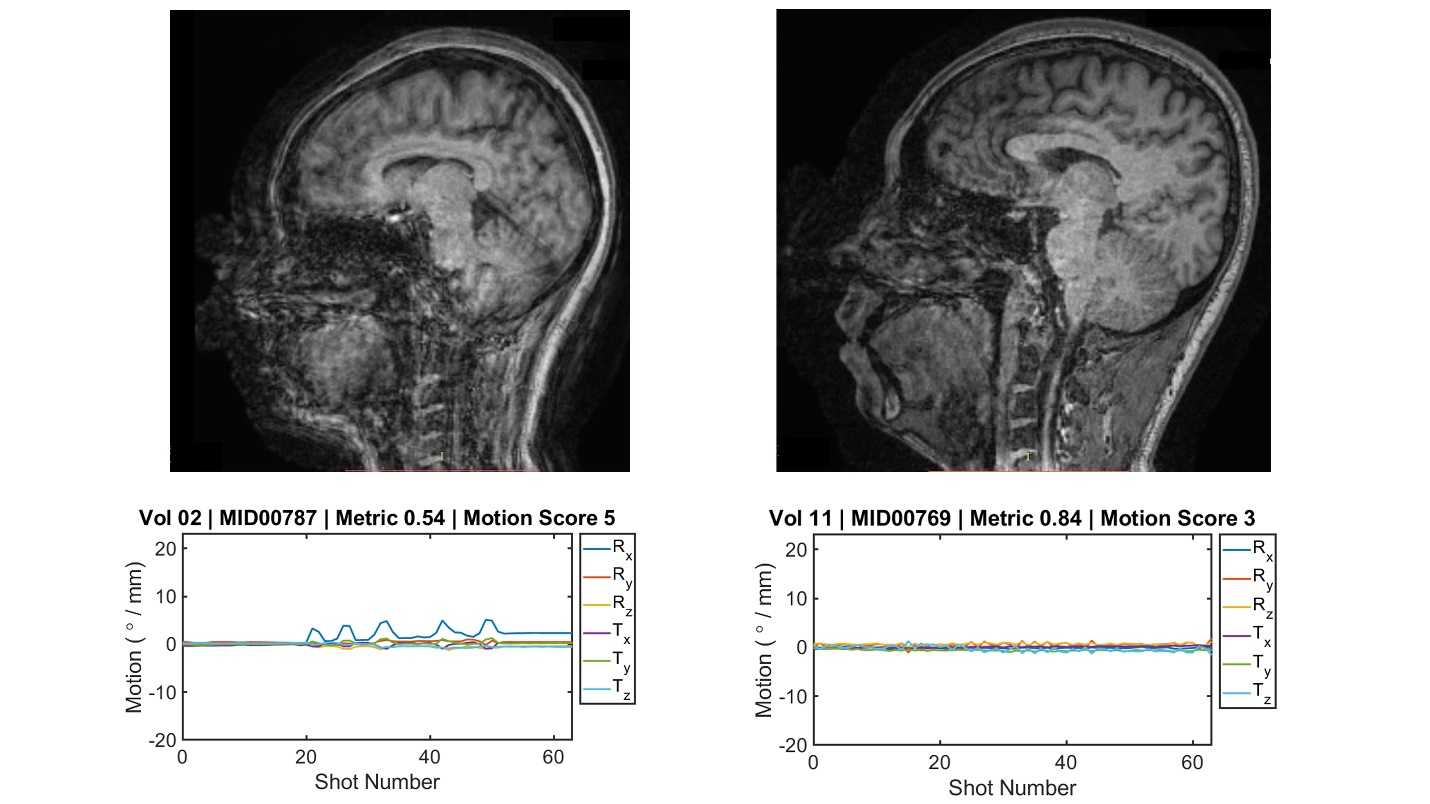


**Supplemental Figure 2.** Individual examples of variation in motion metrics. Volunteer 02 (left) demonstrates qualitatively severe pre-correction motion (Motion Score 5), while volunteer 11 (right) demonstrates mild pre-correction motion (Motion Score 3). However, the overall quantitative motion metric of volunteer 02 (0.54) is lower than that of volunteer 11 (0.84).


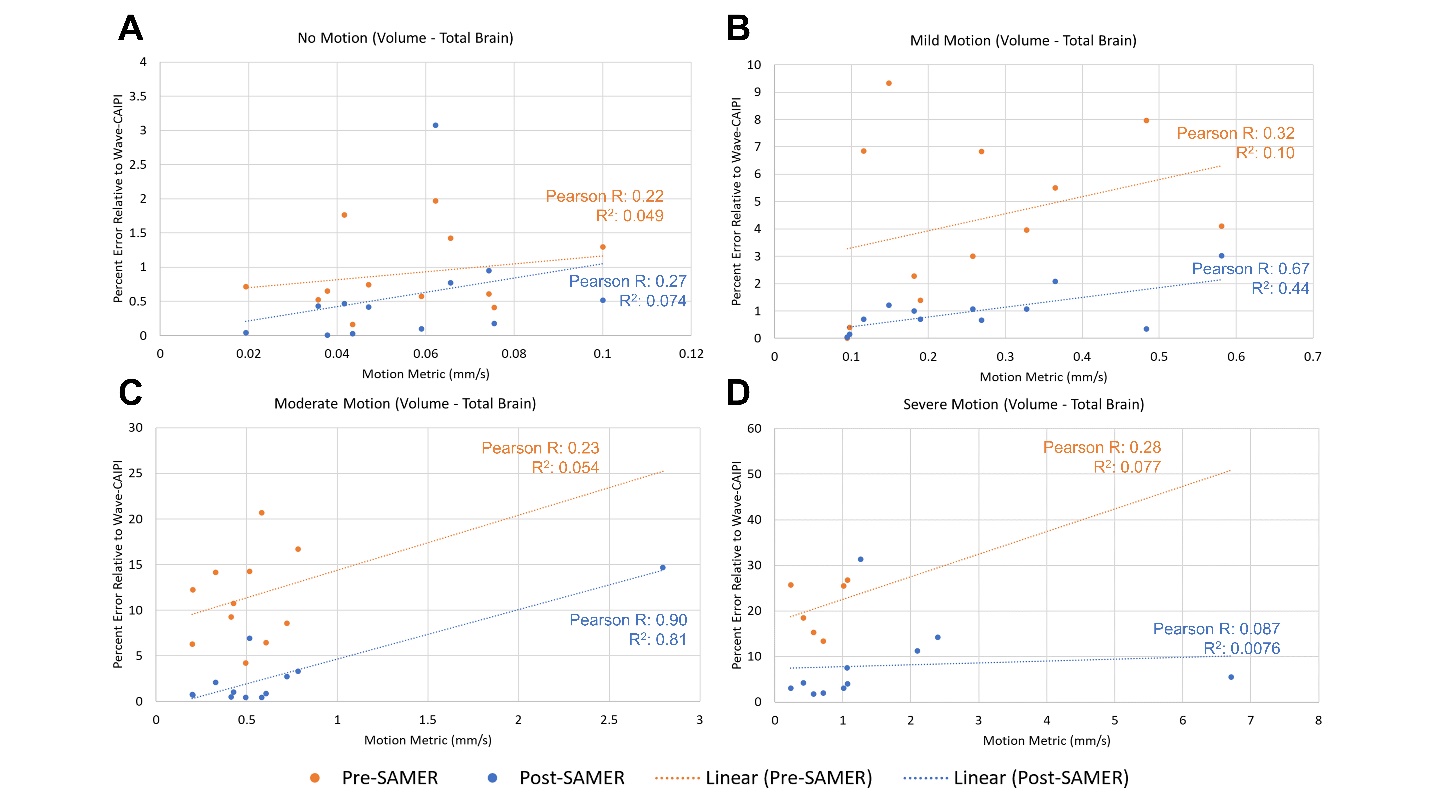


**Supplemental Figure 3.** Linear regression of the percent error relative to the Wave-CAIPI reference standard scan versus quantitative motion metric values in mm/s for the total brain cortical volume for the 12 volunteers, stratified by pre- (orange) and post-SAMER-correction (blue) status for the four different motion states: (A) No, (B) Mild, (C) Moderate, and (D) Severe Motion. Points for which FreeSurfer could not calculate cortical volume are excluded. Pearson correlation and R^2^ values are listed corresponding to the regressions of the pre- (orange) and post-SAMER-correction (blue) data.


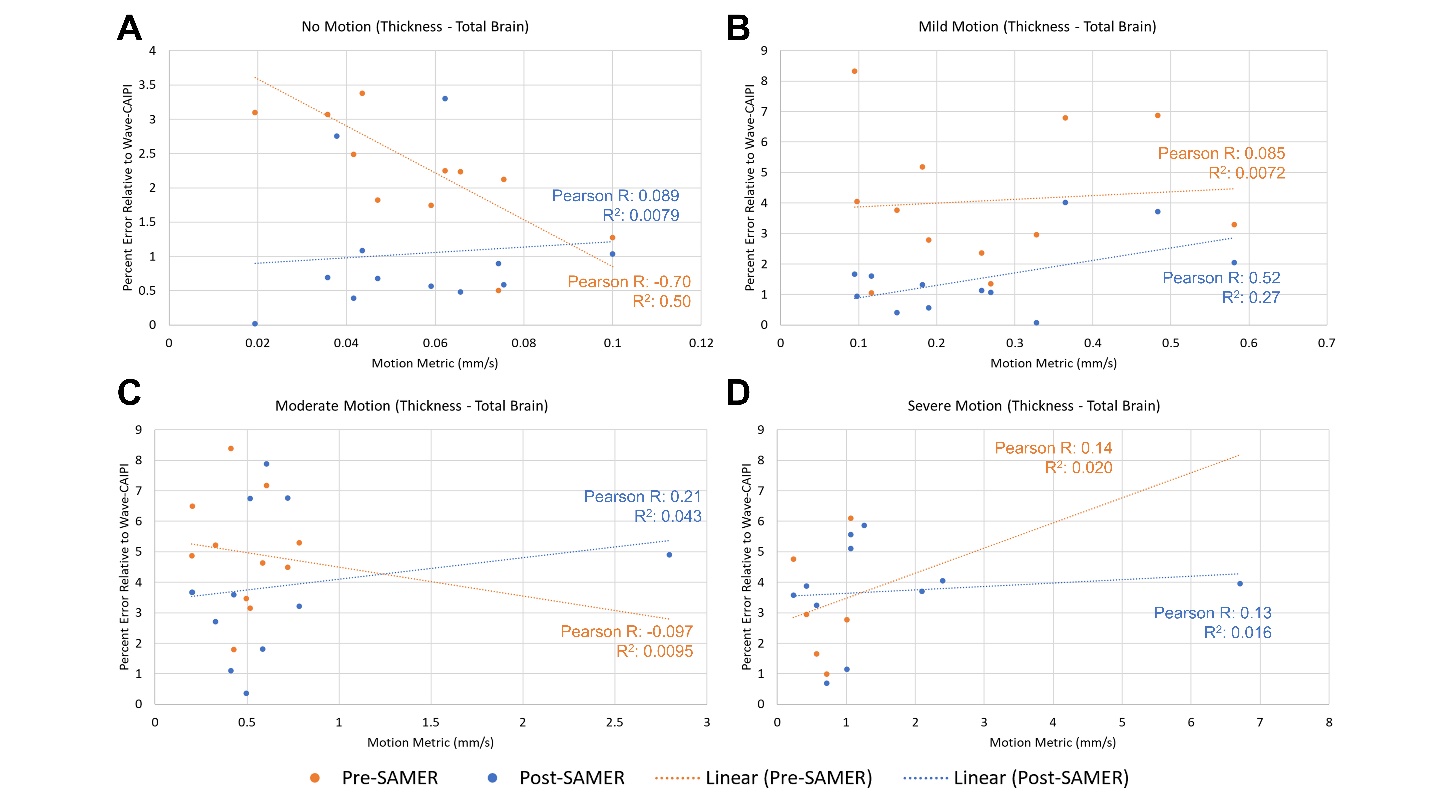


**Supplemental Figure 4.** Linear regression of the percent error relative to the Wave-CAIPI reference standard scan versus quantitative motion metric values in mm/s for the total brain cortical thickness for the 12 volunteers, stratified by pre- (orange) and post-SAMER-correction (blue) status for the four different motion states: (A) No, (B) Mild, (C) Moderate, and (D) Severe Motion. Points for which FreeSurfer could not calculate cortical volume are excluded. Pearson correlation and R^2^ values are listed corresponding to the regressions of the pre- (orange) and post-SAMER-correction (blue) data.


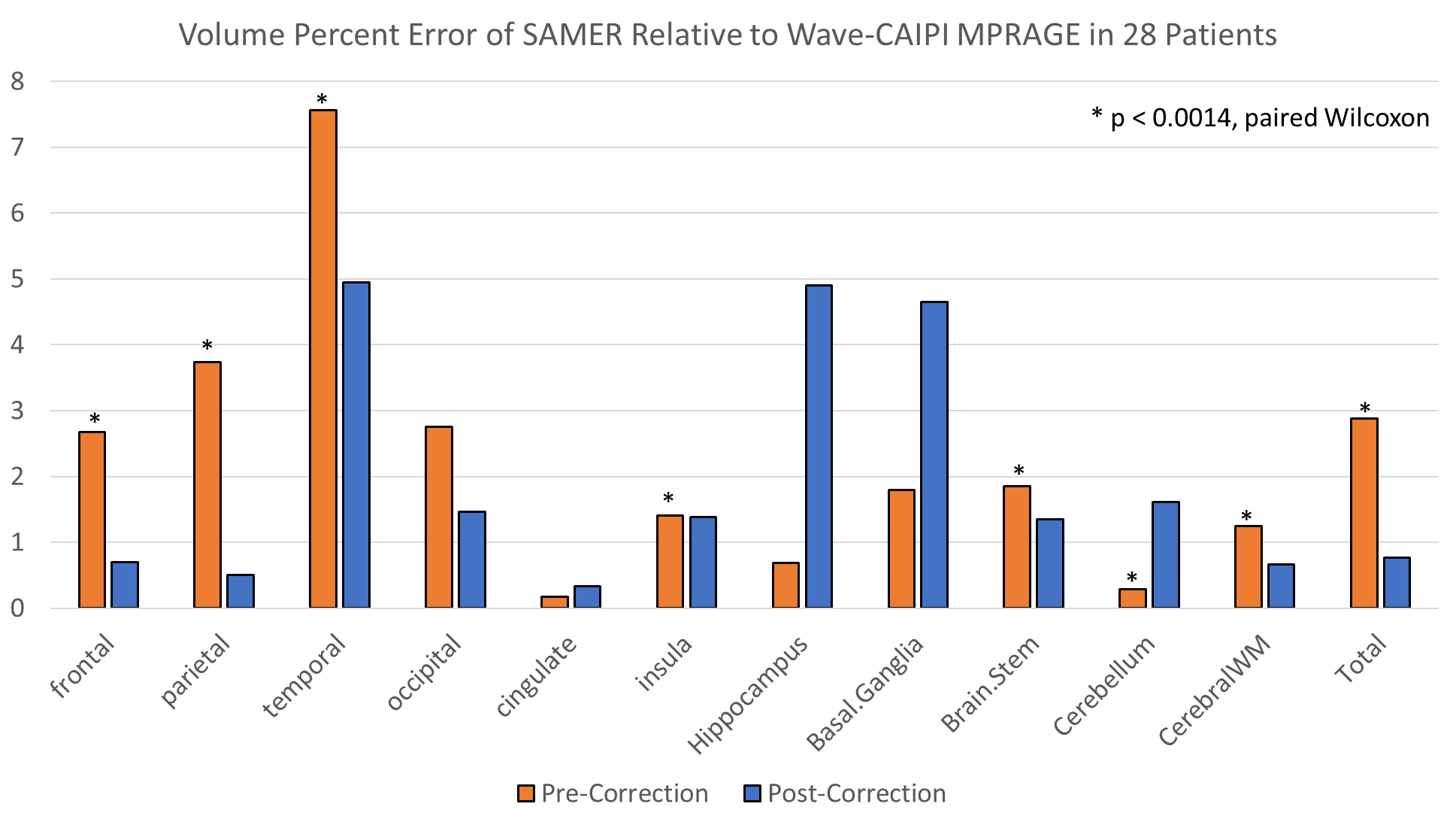


**Supplemental Figure 5.** Median percent error for cortical volume calculations of SAMER scans, pre- and post-correction, relative to Wave-CAIPI MPRAGE scans over 11 anatomical regions and the total brain for the 29 patients in part (2) of our study. A reduction in percent error is seen for most outer brain regions (e.g. the frontal, parietal, temporal, and occipital lobes). On the other hand, there are increases in percent error for some deep brain structures (e.g. the hippocampus and basal ganglia). Statistical significance between volume comparisons (p < 0.0014, Wilcoxon rank sum test) is noted with asterisks (*). Of note, there were no statistically significant differences in volumes between the SAMER post-correction scans and the Wave-CAIPI MPRAGE scans.


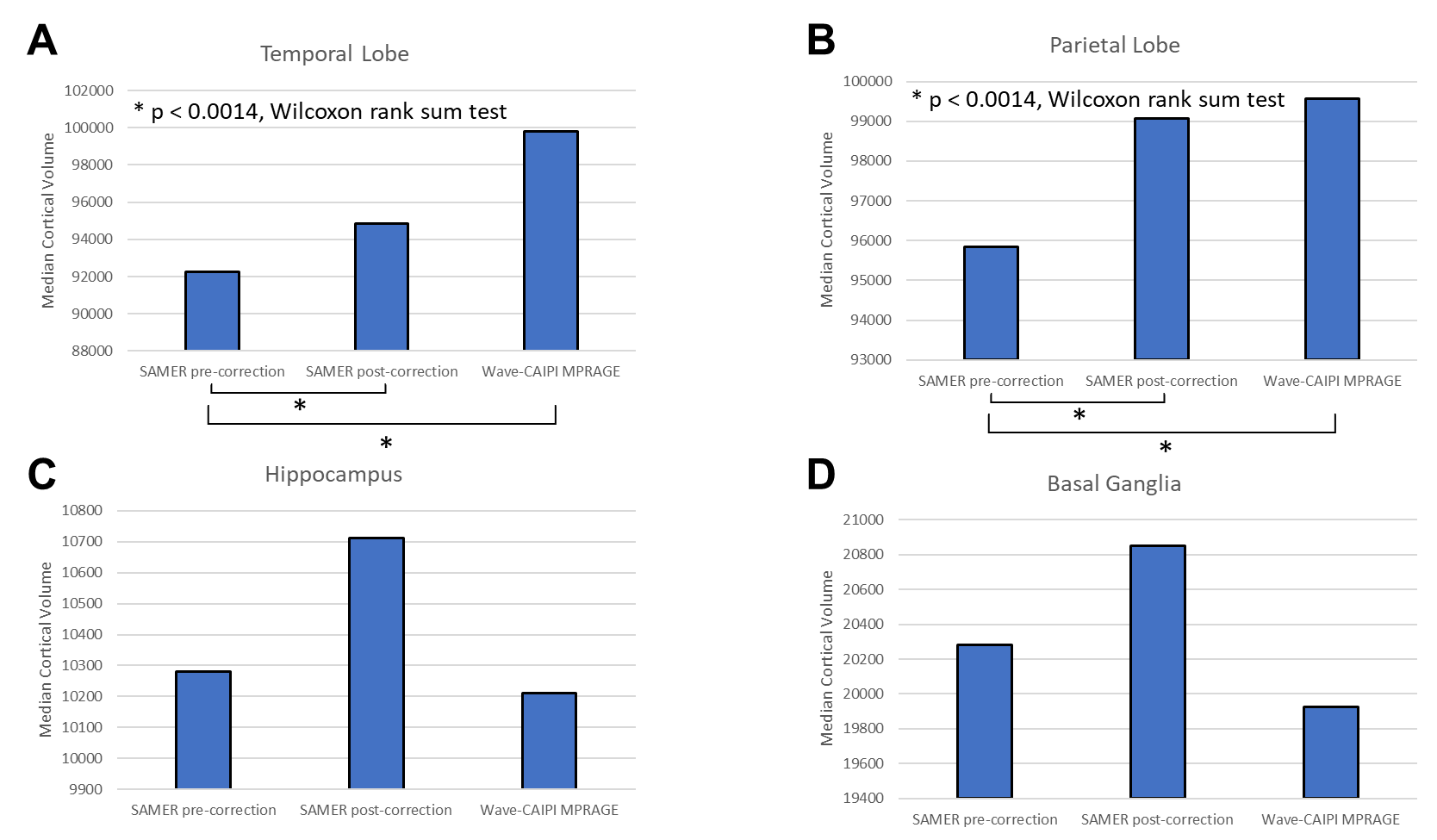


**Supplemental Figure 6.** Comparison of median calculated cortical volumes (mm3) for the SAMER pre- and post-correction scans with Wave-CAIPI MPRAGE for specific anatomical regions in 29 patients. Statistical significance between volume comparisons (p < 0.0014, Wilcoxon rank sum test) is noted with asterisks (*). The temporal (A) and parietal (B) lobes illustrate results representative of most other anatomical regions, with the Wave-CAIPI MPRAGE scans resulting in higher estimated cortical volumes. In contrast, the hippocampus (C) and basal ganglia (D) appear to show lower cortical volumes in their Wave-CAIPI MPRAGE scans. Although the differences are not statistically significant, this is likely the source of why the percent error appears to increase for post-correction scans in these regions.


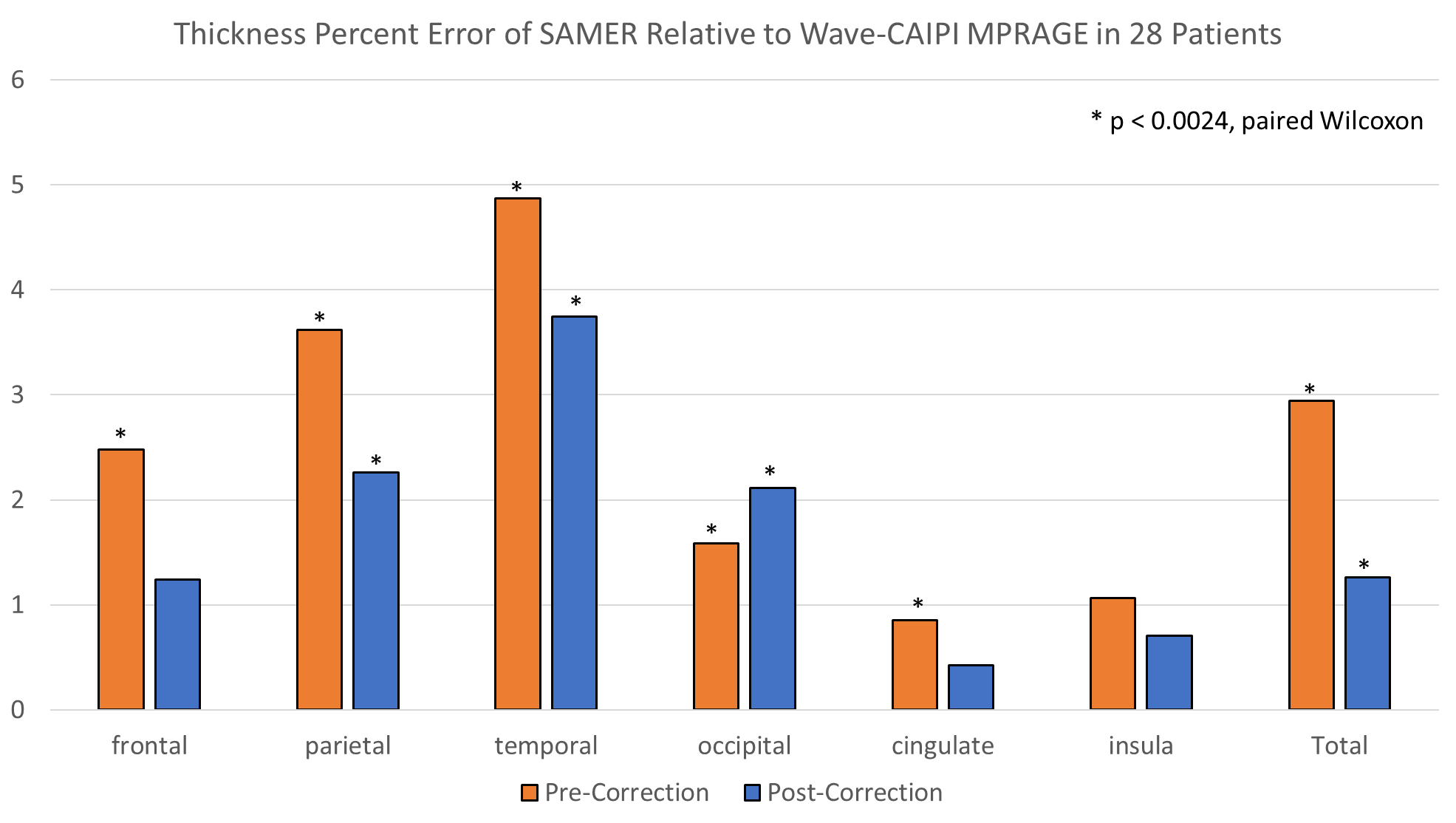


**Supplemental Figure 7.** Median percent error for mean cortical thickness calculations of SAMER scans, pre- and post-correction, relative to Wave-CAIPI MPRAGE scans over 6 anatomical regions and the total brain for 28 patients in part (2) of our study; one subject was excluded due to not having Wave-CAIPI MPRAGE sequences available. Small reductions in percent error are seen for most anatomical regions, most prominently in the frontal parietal, and temporal lobes, as well as over the total brain. Statistical significance between volume comparisons (p < 0.0024, Wilcoxon rank sum test) is noted with asterisks (*).


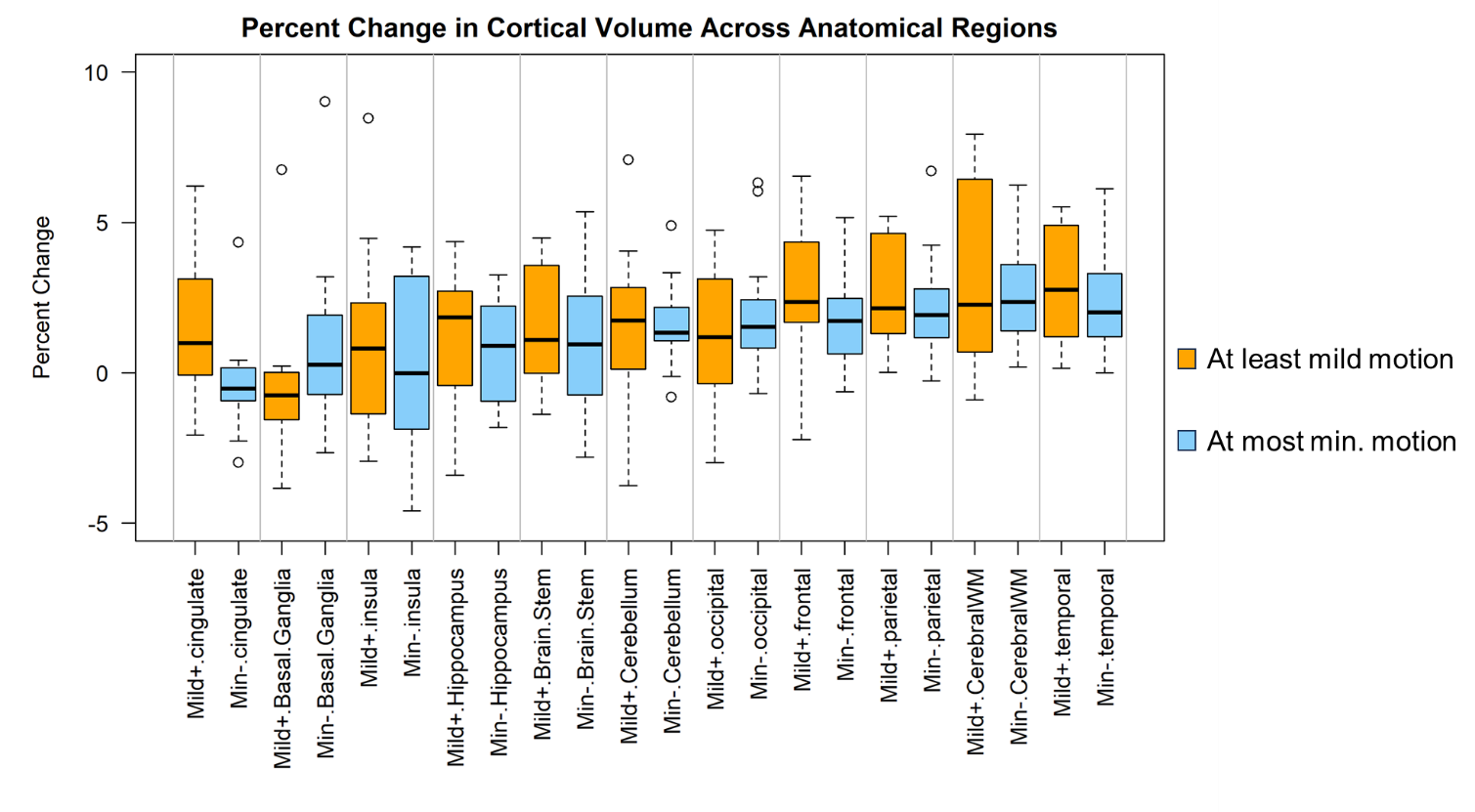


**Supplemental Figure 8.** Percent change in cortical volume across anatomical regions for the patients in part (2) of this work, separated by SAMER pre-correction motion state. 17 patients displayed at least mild motion (mild+) and 12 patients displayed at most minimal motion (min-). The percent changes in cortical volume are similar between the two groups of patients with different motion states. No differences between groups were statistically significant.


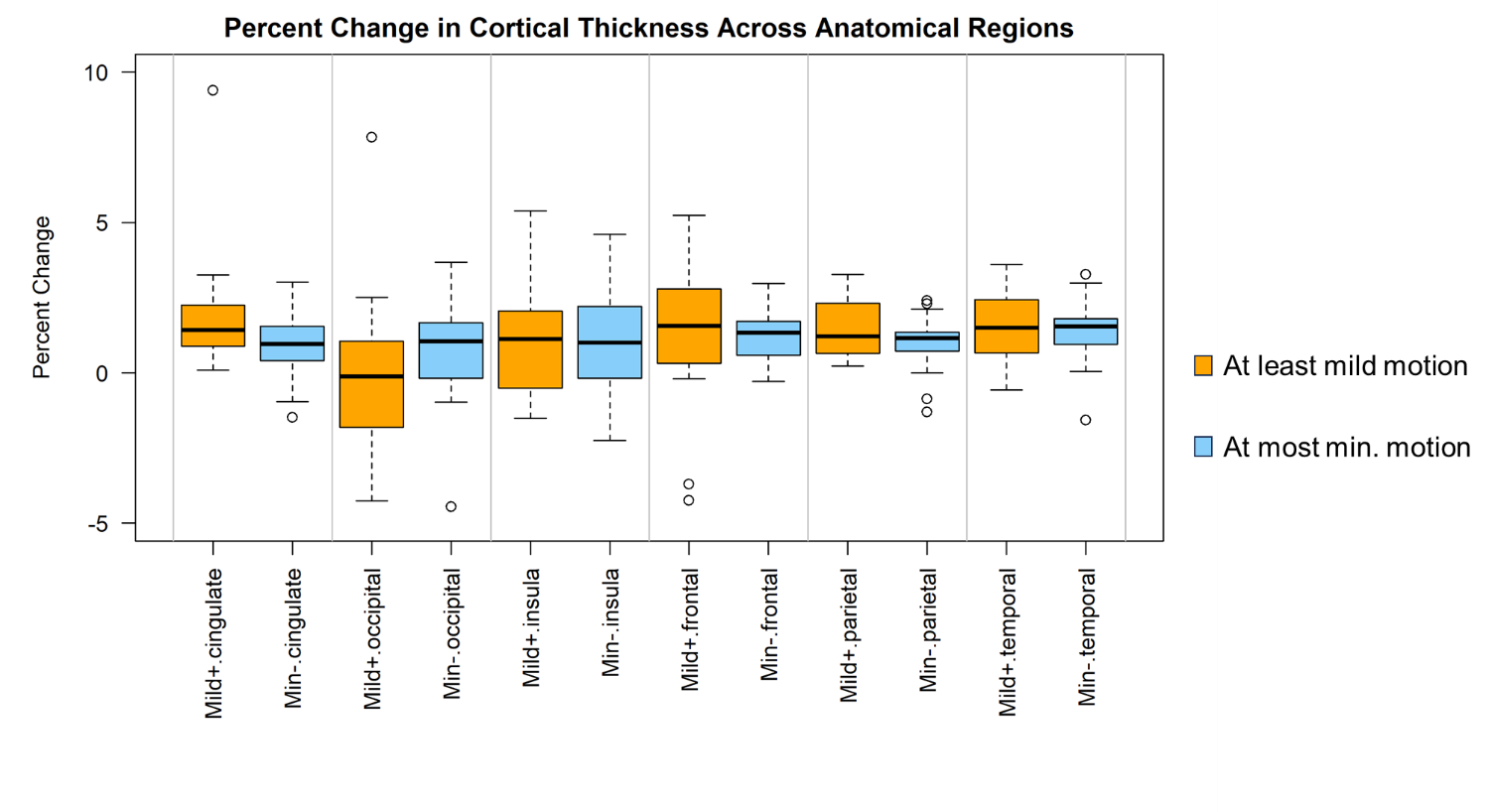


**Supplemental Figure 9.** Percent change in cortical volume across anatomical regions for the patients in part (2) of this work, separated by SAMER pre-correction motion state. 17 patients displayed at least mild motion (mild+) and 12 patients displayed at most minimal motion (min-). The percent changes in cortical thickness are similar between the two groups of patients with different motion states. No differences between groups were statistically significant.

**Supplemental Table 1.** Standardized five-point numerical motion scale used in this work.


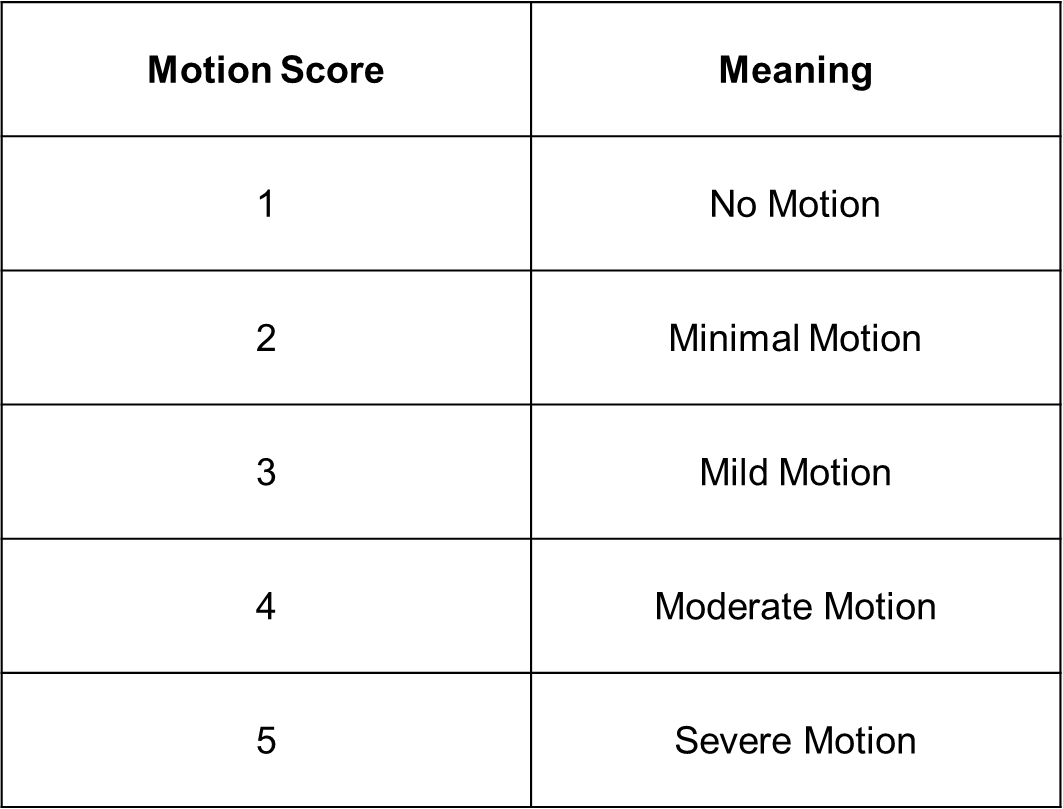


**Supplemental Table 2.** Motion grades for each of the 29 patients in part (2) of the study for SAMER Pre-Corrected scans and Wave-CAIPI-MPRAGE reference scans. The mean SAMER pre-correction motion score was 2.45 and the mean Wave-CAIPI-MPRAGE motion score was 1.89. The Wave-CAIPI-MPRAGE scan for subject 22 was not available for evaluation.


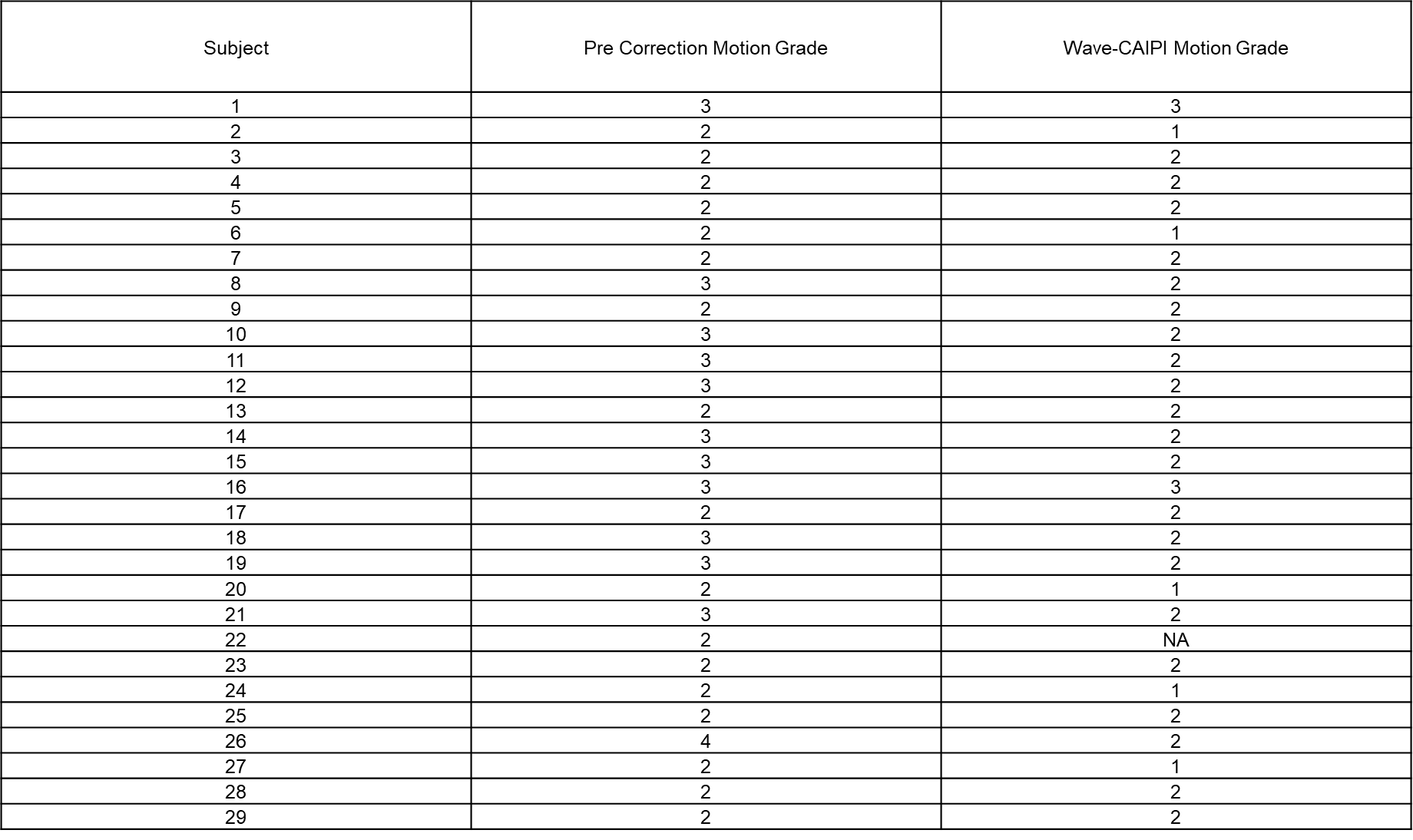


**Supplemental Table 3.** Distribution of SAMER pre-correction motion scores among the 29 patients in part (2) of the present study. Most motion scores were either “minimal” (2) or “mild” (3).


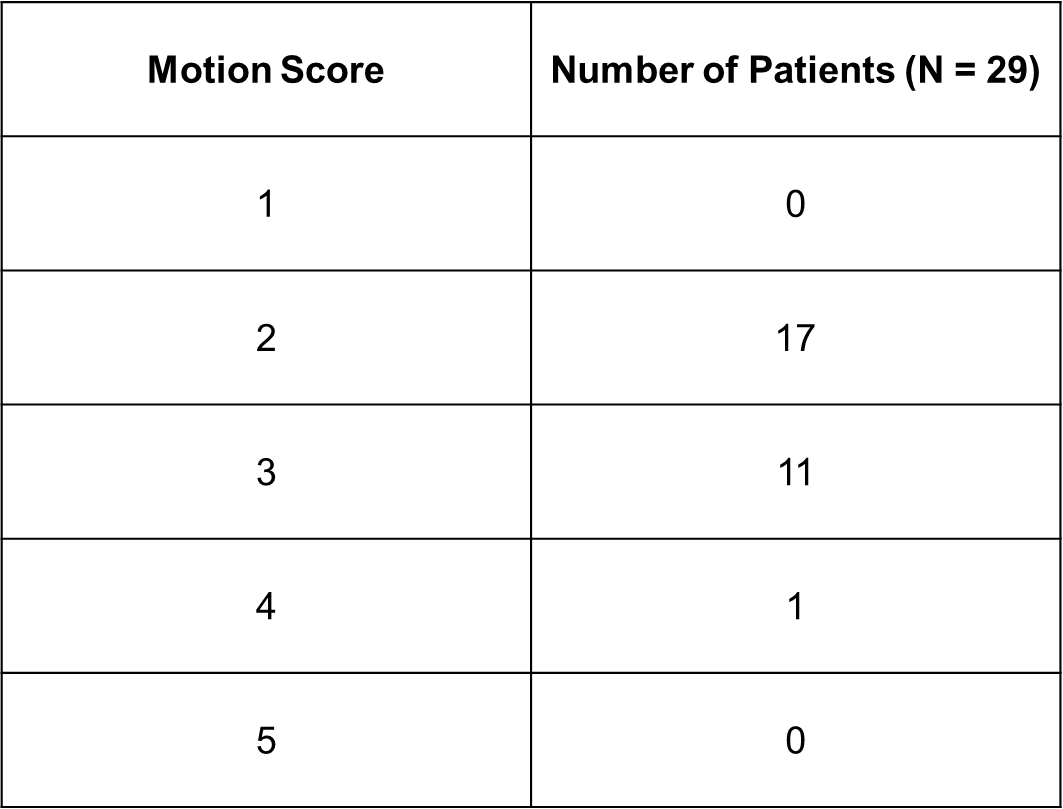

Supplement: 1 [file NIHMS2028451-supplement-1.docx]
